# Circular RNA *circIPO11* drives self-renewal of liver cancer initiating cells via Hedgehog signaling

**DOI:** 10.1186/s12943-021-01435-2

**Published:** 2021-10-14

**Authors:** Yang Gu, Yanying Wang, Luyun He, Jiahang Zhang, Xiaoxiao Zhu, Nian Liu, Jianyi Wang, Tiankun Lu, Lei He, Yong Tian, Zusen Fan

**Affiliations:** 1 CAS Key Laboratory of Infection and Immunity, CAS Center for Excellence in Biomacromolecules, Institute of Biophysics, Chinese Academy of Sciences, Beijing, 100101 China; 2 University of Chinese Academy of Sciences, Beijing, 100049 China; 3 Department of Pathophysiology, School of Basic Medical Sciences, Zhengzhou University, Zhengzhou, 450001 China; 4 CAS Key Laboratory of RNA Biology; Institute of Biophysics, Chinese Academy of Sciences, Beijing, 100101 China; 5 Department of Hepatobiliary Surgery, PLA General Hospital, Beijing, 100853 China

**Keywords:** Liver cancer, cancer initiating cell, circIPO11, Self-renewal, Hedgehog signaling

## Abstract

**Background:**

Hepatocellular carcinoma (HCC) is one of the most intractable tumors in the world due to its high rate of recurrence and heterogeneity. Liver cancer initiating cells also called cancer stem cells (CSCs) play a critical role in resistance against typical therapy and high tumor-initiating potential. However, the role of the novel circular RNA (circRNA) *circIPO11* in the maintenance of liver cancer initiating cells remains elusive.

**Methods:**

CircRNAs highly conserved in humans and mice were identified from 3 primary HCC samples by circRNA array. The expression and function of *circIPO11* were further evaluated by Northern blot, limiting dilution xenograft analysis, chromatin isolation by RNA purification-PCR assay (ChIRP) and HCC patient-derived tumor cells (PDC) models. *CircIpo11* knockout (KO) mice were generated by a CRISPR/Cas9 technology.

**Results:**

*CircIPO11* is highly expressed in HCC tumor tissues and liver CSCs. *CircIPO11* is required for the self-renewal maintenance of liver CSCs to initiate HCC development. Mechanistically, *circIPO11* recruits TOP1 to *GLI1* promoter to trigger its transcription, leading to the activation of Hedgehog signaling. Moreover, GLI1 is also highly expressed in HCC tumor tissues and liver CSCs, and TOP1 expression levels positively correlate with the metastasis, recurrence and survival of HCC patients. Additionally, *circIPO11* knockout in mice suppresses the progression of chemically induced liver cancer development.

**Conclusion:**

Our findings reveal that *circIPO11* drives the self-renewal of liver CSCs and promotes the propagation of HCC via activating Hedgehog signaling pathway. Antisense oligonucleotides (ASOs) against *circIPO11* combined with TOP1 inhibitor camptothecin (CPT) exert synergistic antitumor effect. Therefore, *circIPO11* and the Hedgehog signaling pathway may provide new potential targets for the treatment of HCC patients.

**Supplementary Information:**

The online version contains supplementary material available at 10.1186/s12943-021-01435-2.

## Background

Hepatocellular carcinoma (HCC), a major type of primary liver cancer, is the second leading cause of cancer-related deaths globally [1]. Because of its increasing morbidity and mortality, liver cancer has become a growing global health-care problem [2]. But the effective treatments for liver cancer are limited. Traditional surgical resection, radiotherapy and chemotherapy are prone to disease recurrence, resulting in poor prognosis [3]. New treatments come from an improved understanding of HCC tumorigenesis. Two main biological characteristics of HCC are high recurrence and heterogeneity [4]. Studies have shown that a group of stem/progenitor cells known as cancer initiating cells or cancer stem cells (CSCs) can contribute to organization hierarchy and lead to heterogeneity [5]. Unlike bulk tumor cells, these CSCs display the ability to self-renew, differentiate, and form new tumors, accounting for resistance to traditional treatments and the high recurrence rate of HCC [6]. Recently, plenty of cell surface markers have been identified to separate liver CSCs, such as CD13, CD133, CD90 and EpCAM and so on [7]. However, the mechanism of maintaining liver CSC self-renewal remains elusive.

CSCs are similar to embryonic or adult tissue stem cells in that they rely on activation of highly conserved stemness signaling pathways to maintain their self-renewal and regeneration [8]. Accumulating evidence suggests that alteration of stemness signaling pathways, including Hedgehog (Hh), Wnt and Notch signaling, can promote tumor initiation and progression [9]. Of note, aberrant activation of these pathways in stem cells may evoke uncontrolled cell proliferation and abnormal differentiation, leading to tumor initiation. Moreover, their reactivation can induce tumor reprogramming, resulting in the onset of CSC phenotypes. These stemness pathways also contribute to the hepatocarcinogenesis and recurrence [10]. We previously isolated a rare subset from HCC cells using two surface markers, CD13 and CD133, and defined CD13^+^CD133^+^ cells as liver CSCs, CD13^−^CD133^−^ cells as non-CSCs [11, 12]. Moreover, we also confirmed that several long noncoding RNAs (lncRNAs) can regulate the self-renewal of liver CSCs via Wnt and BMP signaling [13, 14].

Circular RNAs (circRNAs), a class of single-stranded covalent closed RNAs, are generated by backsplicing derived from precursor mRNA (pre-mRNA). As a new class of non-coding RNAs, some circRNAs are highly conserved across species with cell-specific and tissue-specific characteristics. Recent studies have shown that some specific circRNAs that accumulate in the brain are conserved from human to fruit fly [15]. Due to difficultly degraded by RNaseR and lack of polyadenylated (poly A) tails compared with linear RNA, circRNAs are rarely detected in next-generation RNA sequencing (RNA-seq) profiling that is usually enriched in poly(A) + RNA. Until now, most of the well-characterized circRNAs have been identified and established database such as circBase (http://circrna.org/). CircRNAs functions in extensive biological processes have been elucidated by diverse mechanisms. Emerging studies have illustrated that some circRNAs are involved in neuropsychiatric disorder, innate immune responses, hematopoiesis and cell proliferation [16–18]. Interestingly, circ-CDYL, specifically up-regulated in the early stage of HCC, can be used as a potential biomarker for early surveillance of liver cancer [19]. However, it is largely unknown whether circRNAs participate in the regulation of liver CSC self-renewal. The ability of circRNAs to regulate gene expression makes them potential drug targets. Targeting circLONP2 via anti-sense oligonucleotides (ASOs) showed excellent therapeutic effects on colorectal carcinoma [20]. However, whether circRNAs can be used as drug candidates in liver cancer is still worth exploring.

DNA topoisomerases have an essential role in chromatin dynamics, transcription, replication, DNA damage repair and genomic stability via introducing transient DNA breaks for relaxing supercoiled DNA [21]. For example, topoisomerase 1 (TOP1) binds DNA substrates to form a TOP1 cleavage complex (TOP1cc), which causes transient DNA nicks to relax supercoiled DNAs. Camptothecin (CPT) and its derivatives inhibit TOP1 by binding at the enzyme-DNA interface leading to blockade of religation of TOP1cc [22]. Previous studies showed that inhibition of topoisomerases can increase chemosensitivity of HCC cells [23]. However, how topoisomerases function in liver CSCs remains unclear. Here we identified a conserved circRNA *circIPO11* (originated from *IPO11* gene transcript, circbase symbol hsa_circ_0007915) that is highly expressed in tumor tissues and liver CSCs. *CircIPO11* associates with TOP1 to initiate GLI1 transcription and activates Hh signaling for self-renewal maintenance of liver CSCs. Moreover, combination administration of ASOs against *circIPO11* and TOP1 inhibitor CPT has effective therapeutic effect on HCC tumors.

## Materials and methods

### Antibodies and reagents

Anti-histone H3 (catalog 4499) antibody was purchased from Cell Signaling Technology. Anti-EEA1 (catalog sc-6415) antibody was from Santa Cruz Biotechnology. Anti-TOP1 (catalog ab109374) antibody was from Abcam. Anti-β-actin (catalog A1978), anti-Flag (catalog F1804) antibodies were from Sigma-Aldrich. Anti-GLI1 (catalog 66,905–1-Ig), anti-IPO11 (catalog 14,403–1-AP), anti-SMO (catalog 20,787–1-AP), anti-CD13 (catalog 14,553–1-AP) and anti-Nanog (catalog 14,295–1-AP) antibodies were purchased from Proteintech Group Inc. Anti-PTCH1 (catalog LS-C114391) antibody was from LifeSpan BioSciences. HRP-conjugated secondary antibodies were from Sungene Biotech. FITC-conjugated CD13 antibody (catalog 11–0138) was obtained from eBioscience. PE-conjugated CD133 (catalog 130–098-826) was from Miltenyi Biotec. Alexa405, Alexa488 and Alexa594-conjugated secondary antibodies were obtained from Invitrogen. bFGF (catalog GF446-50UG) was obtained from Millipore. EGF (catalog E5036-200UG) and DAPI (catalog 28,718–90-3) were from Sigma-Aldrich. N2 supplement (catalog 17,502–048) and B27 (catalog 17,504–044) were obtained from Invitrogen. The Dual Luciferase Reporter Gene Assay Kit (catalog RG027) was purchased from Beyotime. D-Luciferin (catalog 40902ES01) was obtained from Yeasen BioTechnologies. Biotin RNA Labeling Mix (catalog 11,685,597,910) and T7 RNA polymerase (catalog 10,881,767,001) were from Roche. The LightShift™ Chemiluminescent RNA EMSA kit (catalog 20,158) and Chemiluminescent Nucleic Acid Detection Module (catalog 89,880) were from Thermo Scientific. Ultra-low attachment plates (catalog 3471) were purchased from Corning 70μm company.

### Cell lines and HCC samples

Human HCC cell lines Huh7 and PLC/PRF/5 (PLC) were provided by Dr. Zeguang Han (Shanghai Jiao Tong University School of Medicine, Shanghai, China). All these cell lines were maintained in DMEM medium supplemented with 10% FBS, 100 μg/ml penicillin G, and 100 U/ml streptomycin. Human HCC samples were obtained from consenting patients at the Department of Hepatobiliary Surgery, PLA General Hospital (Beijing, China). HCC samples were treated within 2 h after resection. Necrotic tissue was removed, and tumor was cut into 1 mm^3^ with scissors. Then digested with collagenase IV for 45 min (0.05% collagenase IV, 0.05% proteinase, 0.01% DNase), shaken every 15 min. A 70 μm cell filter was used and centrifuged at 50 g for 1 min. Supernatants were collected and centrifuged at 150 g for 8 min. And HCC cells were enriched at the bottom. After red cell elimination, HCC primary cells were obtained and used for sphere cultivation. Oncosphere cells were cultured in DMEM/F12 serum-free medium supplemented with 20 ng/ml bFGF, 20 ng/ml EGF, N2 and B27.

### Mouse strains

*CircIpo11*^−/−^ mice were generated using CRISPR/Cas9 approaches [24]. The intronic sequence between exon 3 and exon 4 of Ipo11 gene was targeted by sgRNAs (Table S2). After confirming cleavage efficiency of these sgRNAs in 293 T cells, in vitro transcribed mRNAs containing the sgRNA/Cas9 sequences were injected into zygotes through microinjection. Approximate 200 ~ 250 zygotes in C57BL/6 N background were implanted into the uterus of surrogate mice, from which F0 mice were generated. Genotypes of F0 mice were subsequently verified by PCR and DNA sequencing. Identification of primers were listed in Table S3. WT allele had a PCR length of about 222 bp and deficient allele had a PCR length of about 336 bp. F0 mice were crossed to generate *circIpo11*^−/−^ mice. BALB/c nude mice were from Beijing Vital River Laboratory Animal Technology. Immunodeficient B-NDG mice were from Biocytogen. Mice were sacrificed when they developed tumors larger than 15 mm in diameter or skin ulceration. Mice were fed under specific pathogen-free (SPF) grade with approval by the Institutional Animal Care and Use Committees at the Institute of Biophysics, Chinese Academy of Sciences. For DEN-induced liver tumors model, Mice were injected intraperitoneally with 25 mg/kg of diethylnitrosamine (DEN, Sigma, 73,861) at 2 weeks old. Livers were collected and analyzed after indicated times of DEN injection.

### CRISPR/Cas9 knockout system

*GLI1*, *TOP1,* and *GLI1* promoter-deletion cells were established using CRISPR/Cas9 approaches provided by Zhang’s lab [13]. All sgRNAs were designed by online CRISPR Design Tool (http://crispr.mit.edu/). In brief, for *GLI1* or *TOP1* targeting, sgRNAs were designed to target the fourth exon of *GLI1* (exon 4) or *TOP1* (exon 3), and cloned into LentiCRISPRv2 (puro, catalog 52,961, Addgene). We used LentiCRISPRv2, pVSVg (catalog 8454, Addgene), and psPAX2 (catalog 12,260, Addgene) plasmids to produce CRISPR–Cas9 lentivirus. For *GLI1*-deleted and *TOP1*-deleted cells, single sgRNA was used. For *GLI1* promoter-deleted cells, a pair of sgRNAs targeting sequences flanking the binding sequence (− 2050 bp ~ − 1850 bp) was used. sgRNA sequences targeting *GLI1*, *TOP1,* and *GLI1* promoter were listed in Table S2. We generated lentivirus in 293 T with lipo3000 for 2 days. After the supernatant was filtered with 70μm strainer and mixed with equal volume fresh DMEM (10%FBS), HCC cells were infected for 12 h, followed by puromycin selection and monoclonalization. The monoclines were propagated and confirmed by DNA electrophoresis and sequencing. The correctly targeted clones were obtained and used for subsequent experiments. For rescue assay, *circIPO11* or *GLI1*–overexpressing lentivirus was co-infected, followed by GFP sorting. Finally, GLI1 KO CSCs and non-CSCs were isolated by FACS for further research.

### Overexpression of *circIPO11,* Δ*circIPO11* and GLI1

For *circIPO11* overexpression, the exonic sequences of *circIPO11* (Exon4 and 5 of *IPO11* gene) and complementary sequences were needed. The intronic sequences including complementary sequences were obtained from genomic DNA. Then the full sequences were cloned into the pBPLV-GFP vector. For Δ*circIPO11* overexpression, the TOP1-binding region (31-94 nt) was deleted. For GLI1 overexpression, pBPLV-GFP-GLI1 plasmids were constructed. Then these plasmids and their package plasmids (pBPLV: VSVG: pLp1: pLp2 = 5: 2.8: 4.2: 2μg) were transfected into 293 T cells for virus generation. After supernatants was filtered with 70 μm strainer and mixed with equal volume fresh DMEM (10%FBS), HCC cells were infected for 12 h, followed by GFP sorting.

### Immunohistochemical staining

Immunohistochemical staining was performed as previously described [11]. In brief, paraffin-embedded tumor tissue sections were deparaffinized in xylene and rehydrated in graded alcohol (100, 100, 95, 85, and 70% alcohols). The slides were processed for antigen retrieval in Tris-EDTA buffer (10 mM, pH 8.0), 100 °C for 15 min, and then cooled down naturally. Treatment with 3% hydrogen peroxide (H_2_O_2_) for 10 min blocked the activity of endogenous peroxidase. After blocking with 10% donkey serum for 30 min, the sections were incubated overnight at 4 °C with the primary antibodies. After washing with PBS, HRP-conjugated secondary antibodies were incubated at RT for 1 h. Subsequently detection was performed using DAB, counterstained with hematoxylin, dehydrated and mounted.

### IVIS in vivo imaging

IVIS in vivo imaging was performed as described [14]. In brief, anaesthetized B-NDG mice and open the abdominal cavity to expose the liver. 1 × 10^6^ luciferase transfected Huh7 cells were in situ injected into the liver with insulin needle. The wound was sutured and coated with antibiotics, and fed with acidified water (PH3.0). After 2 weeks, D-fluorescein potassium salt was injected intraperitoneally of the mice. After 5 min, the mice were anesthetized with isoflurane. Then the luciferase activity was detected in the IVIS-100 imaging system. The collected images are analyzed using Living Image 4.3 software (PerkinElmer).

### Chromatin immunoprecipitation (ChIP) assay

ChIP assay was performed according to the protocol (Uptate Biotechnology, Inc.). In brief, oncosphere cells were treated with 1% formaldehyde for 10 min at 37 °C, and then lysed by SDS buffer for 10 min on ice, followed by ultrasonic (30% of maximum power value) to get 200 ~ 1000 bp DNA fragments. The samples were precleared with salmon sperm DNA/protein agarose beads for 0.5 h in rotor at 4 °C, and then incubated with the anti-TOP1 antibodies overnight. The enrichments after elution were analyzed by qPCR. Primer sequences are shown in the Table S3.

### Chromatin isolation by RNA purification-PCR assay (ChIRP)

ChIRP assay were performed as described [25]. In brief, cross-linked with 1% glutaraldehyde, oncosphere cells were cracked by SDS buffer for 10 min on ice. Subsequently chromatins were sonicated into fragments 200 ~ 1000 bp in length. Antisense probes for *circIPO11* were designed by Biosearch Probe Designer (targeting junction sequence of *circIPO11*). Two sets of biotin-labeled probes (Probeset-circ and Probeset-Ctrl) were added into samples for incubation at 4 °C overnight. Combinative chromatin fragments were enriched by Streptavidin C1 magnetic beads before being purified for qPCR examination.

### Immunofluorescence staining

Fixed by 4% paraformaldehyde (PFA) for 20 min, HCC cells were permeated by 1% triton X-100 for 30 min. After blocking with 10% donkey serum for 30 min, primary antibodies were added and incubated 2 h at 37 °C. After washing with PBS, fluorescence-conjugated secondary antibodies were added and incubated at 37 °C for 1 h. After sealing, confocal microscopy (Nikon A1R+) was performed for observation.

### Measurement of *circIPO11* copy numbers

The linearized plasmid pcDNA3-*circIPO11* was continuously diluted to generate a standard curve of *circIPO11* by qRT-PCR. The copy number of the diluted pcDNA3-*circIPO11* was analyzed via DNA/RNA Copy Number Calculator from website (http://endmemo.com/bio/dnacopynum.php). To measure the *circIPO11* copy number in spheres and CSCs, total RNA extracted from 3 × 10^5^ cells of each HCC line was reverse transcribed into cDNAs, followed by qPCR analysis. The copy number could be measured from the standard curve.

### Cell proliferation assay

2000 ~ 5000 indicated cells were transplanted into a well of 6-well plates and cultured in a cell incubator until the cells grew to 75%. After washing with PBS, the cells were fixed with 4% PFA for 10 min, and incubated with 0.1% crystal violet for 30 min at 37 °C. Plates were washed gently with distilled water, and placed in the oven to air-dry. After photographed, 33% acetic acid was added to each well for decolorization, and then the absorbance value was measured at 570 nm after sufficient shaking.

### Sphere formation assay

Oncospheres were cultured as previous described [11]. Indicated 2000 HCC cells or 5000 HCC primary cells were grown in low attachment 6-well plates and cultured in sphere formation medium (DMEM/F12 supplemented with 20 ng/ml EGF, 20 ng/ml bFGF, B27 supplement, N2 supplement). Numbers of spheres were counted under an optical microscope after 1 ~ 2 weeks and photographs were taken. For non-sphere cell separation, we collected cell culture containing non-sphere cells and sphere cells, and let stand for 10 min. Pellets were sphere cells, and supernatants were non-sphere cells. The sphere cells were collected for subsequent experiments. Supernatants were then collected by centrifugation at 2000 g for 10 min. Pellets were non-sphere cells and used directly for subsequent assays.

### Generation of PDC models

Mouse PDC models were generated as described [26]. For tumor-initiating assays, gradient dilutions (10, 10^2^, 10^3^, and 10^4^) of indicated Huh7 cells were injected with 100 μl Matrigel subcutaneously into the BALB/c nude mice. The percentages of tumor-free mice were calculated 3 months later. Tumorigenic cell frequencies in *circIPO11*-depleted and overexpressed tumor cells were analyzed with a limiting dilution assay (http://bioinf.wehi.edu.au/software/elda/). Eight parallel groups were used per sample. For tumor-formation assays, xenografts were produced by injecting 1 × 10^6^ oncosphere cells subcutaneously into BALB/c nude mice. Tumor sizes were measured every 4 days. Five mice were used per sample. Twenty-five milligram per kilogram ASOs were injected around the tumor every 2 days (16, 18, 20, 22, and 24). Mice were killed at 35th day after HCC primary cells injection.

### RNA pulldown and mass spectrometry assay

Biotin-labeled RNAs (*circIPO11* junction sequences, antisense and IPO11 intron sequences) were transcribed with the T7 RNA polymerase and biotin RNA labeling mix. For RNA pulldown assay [13], probes were incubated with HCC oncosphere lysates overnight at 4 °C. Then the streptavidin-conjugated agarose beads were added for enrichment. After washed with RIPA buffer, biotin-enriched proteins were separated by SDS–PAGE, and visualized by silver staining. Differential bands in SDS-PAGE gels were collected for mass spectrometry (LTQ Orbitrap XL, Thermo).

### Flow cytometry

Isolation of liver CSCs was performed as previously described [12]. Briefly, HCC sample CSCs were labeled with FITC-conjugated CD13 and PE-conjugated CD133 antibodies and isolated by flow cytometry. To analyze survival and proliferation rates of CSCs, Ki-67(17–5699-41, eBioscience), AnnexinV-APC and 7-AAD apoptosis detection kit (AO2001-11A-H, Sungene Biotech) were used according to the manufacturer’s instructions. FlowJo 7.6 software was used for subsequent analysis.

### CircRNAs sequencing and microarray assay

For circRNAs sequencing, RNA was isolated with Trizol method from HCC primary tumor and peri-tumor tissues, and purified with RNease Mini kit. Then circRNA were sequenced using human circRNA Array (8x15K, Arraystar) was performed. For identification of circRNA downstream target genes, total RNA was isolated with Trizol from *circIPO11*-depleted or control oncosphere cells. Then NimbleGen microarray analysis was performed.

### RNA EMSA assay

EMSA experiments were performed using a LightShift Chemiluminescent RNA EMSA Kit (Thermo Scientific). Recombinant TOP1 protein was obtained by purification (Constructed into plasmids pGEX-6P-1 and 3xFlag). RNA fragments of *circIPO11* were transcribed with T7 and labeled with biotin according to standard protocols. Probes and recombinant proteins were incubated in binding buffer and mobility shift assay was performed using native gel electrophoresis.

### DNase I sensibility assay

Cell nuclei were extracted according to the protocol from a nuclei-isolation kit (Sigma-Aldrich). Then cell nuclei were digested with 1 U DNase I for 5 min at 37 °C. After stopping the digestion, total DNA was extracted and followed by qRT-PCR.

### Northern blot

Total RNA was extracted from indicated samples using Trizol, then subjected to electrophoresis on 2% denaturing agarose gel with 1% formaldehyde for 1.5 h. Samples were transferred to positively charged NC membranes with 20 × SSC buffer. After UV cross-linking (265 nm ultraviolet with energy of 200,000 μJ/cm2) and prehybridization, membranes were incubated with biotin-labeled probes at 65 °C for 16 ~ 20 h. After washed with washing buffer, biotin signals were detected with Chemiluminescent Nucleic Acid Detection Module according to the manufacturer’s instructions. For detecting circRNAs only, junction sequences were used for probes.

### Psoralen photobinding assay

Psoralen photobinding assay was performed as described. Briefly, the CPT was added into oncosphere cells, and then treated with psoralen at 37 °C for 8 min. Cross-linked with UV, the oncosphere cells were collected and lysed at 55 °C for 5 h. The DNA was extracted, and followed by ultrasonic to get DNA fragments. The cross-linked and uncross-linked segments were separated by PCR, and then analyzed by qPCR.

### Statistics

Data were analyzed with an unpaired Student’s t-test using the GraphPad Prism 8 software and Excel 2016. *p* < 0.05 was considered significant (*p < 0.05; **p < 0.01; ***p < 0.001); NS, non-significant. For survival analysis, the Kaplan-Meier survival analysis was used. All flow cytometry data were analyzed with FlowJo 7.6.

## Results

### *CircIPO11* is highly expressed in HCC tumors and liver CSCs

**Fig. 1:**
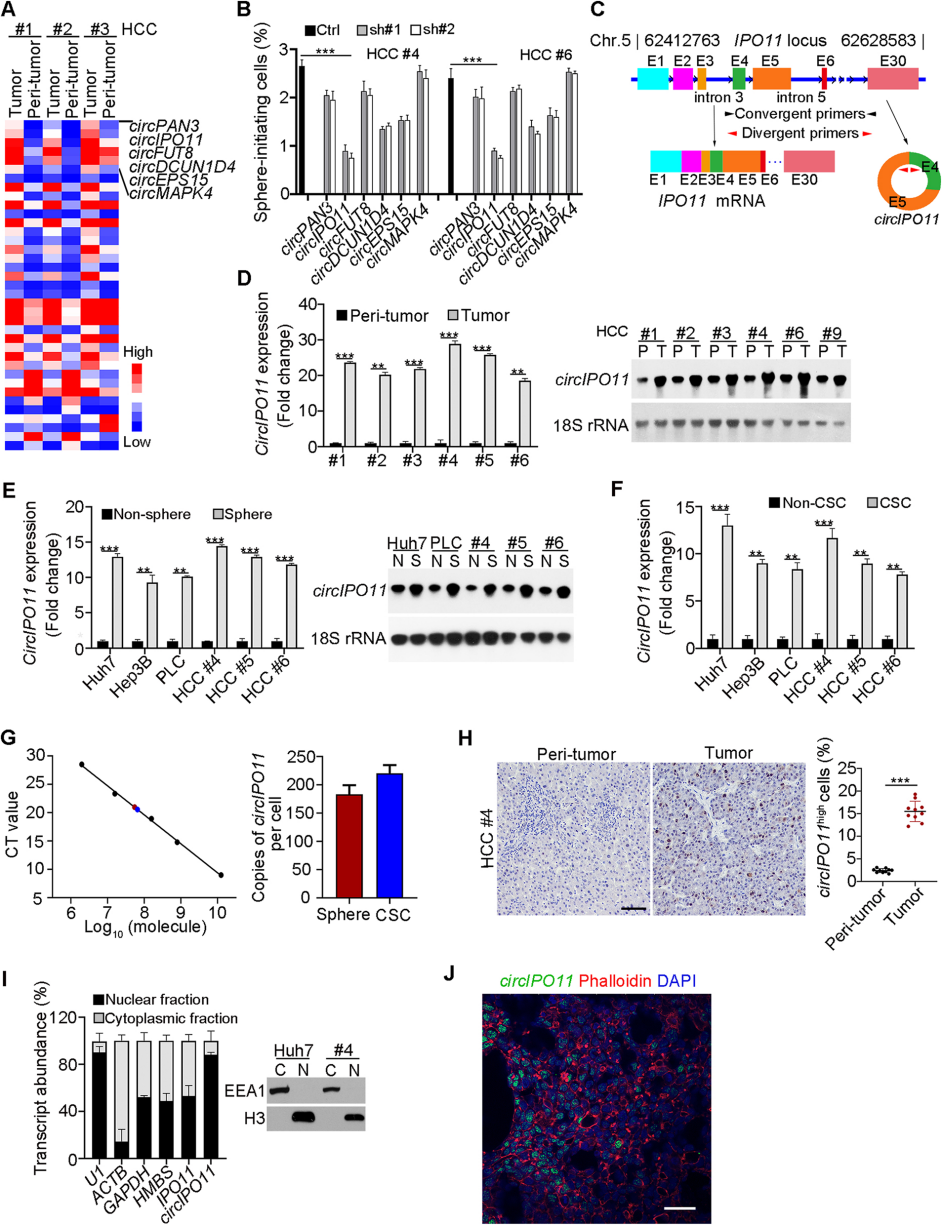
*CircIPO11* is highly expressed in HCC tumors and liver CSCs. **A** Heat map of 46 differentially expressed circRNAs (*P* < 0.05) as well as highly conserved in human and mouse. HCC #1, HCC #2, and HCC #3 denoted HCC sample numbers. Top 6 upregulated circRNAs in tumor were shown. **B** Top 6 upregulated circRNAs in HCC primary cells were depleted using shRNA. Their proportions of sphere formation were measured. Data are shown as means ± SD. **C** Schematic representation of human *circIPO11*. Convergent primers for linear IPO11 were denoted in black arrowheads, and divergent primers specifically for *circIPO11* were denoted in red arrowheads. E1, exon #1. (**D, E**) Expression levels of *circIPO11* in HCC primary tumors and peri-tumors (**D**), or in oncosphere and non-sphere cells (**E**). Data of qRT-PCR were normalized to endogenous 18S rRNA unless noted in this study. Results are shown as means ± SD. Isoforms and size of *circIPO11* were detected by Northern blot, using 18S rRNA as a loading control (right panel). **F** Expression levels of *circIPO11* in liver CSCs (CD13^+^CD133^+^) and non-CSCs (CD13^+^CD133^+^) sorted from liver cancer cell lines and HCC primary cells. **G** Copy numbers of *circIPO11* were analyzed by qRT-PCR. Black dots represent known copies of *circIPO11* from a plasmid pcDNA3 containing *circIPO11* sequences (left), and red and blue dots represent *circIPO11* copies in spheres and liver CSCs (right). Average copies of *circIPO11* per cell were calculated (right). **H** In situ hybridization of *circIPO11* in human HCC tumor and peri-tumor tissues. Representative images are shown in the left panel. Quantitation of *circIPO11* positive cells in sections from 10 different HCC samples was shown in the right panel using Image-Pro Plus 6. Results are shown as means ± SEM. Scale bar, 50 μm. **I** Nuclear-cytoplasmic separation assays were performed using HCC oncosphere cell lysates, followed by qRT PCR (left panel) and Western blot analysis (right panel). U1 RNA served as a positive control for nuclear location. EEA1, endosome antigen 1; H3, histone 3. Data are shown as means ± SD. **J** Representative immunofluorescence staining of *circIPO11* in HCC tumor tissues (*n* = 3). *CircIPO11* was visualized by RNA FISH, followed by Alexa594-conjugated phalloidin (1:3000, 23,122, aatbio). Scale bar, 50 μm. **P < 0.01; *** *P* < 0.001 by two-tailed Student’s t-test. Data are representative of at least three independent experiments

To identify the function circRNAs in the tumorigenesis of HCC, we conducted circRNA transcriptome analysis of human HCC tumor and peri-tumor tissues with human circRNA array (Arraystar). Of note, only a small fraction (10–20%) was highly conserved between humans and mice [27]. We further screened out 46 differentially expressed circRNAs as well as highly conserved in humans and mice (Fig. 1A), including 34 upregulated and 12 downregulated circRNAs. Top six upregulated circRNAs in HCC tumors were listed on the right (Fig. 1A). Biological characteristics of these circRNAs were further validated by sequencing (Fig. S1A), PCR (Fig. S1B), RNase R digestion (Fig. S1C) and actinomycin D treatment (Fig. S1D). In order to explore the roles of these circRNAs in liver CSCs, we depleted these circRNAs in HCC samples and analyzed their sphere formation capacity. Among these circRNAs, *circIPO11* depletion significantly decreased the ability of oncosphere formation (Fig. 1B). C*ircIPO11* was highly expressed in liver cancer tissues by a transcriptomic sequencing [28], which is consistent with our results. *CircIPO11*, located on human chromosome 5, consists of the fourth and fifth exons of *IPO11* gene (Fig. 1C). Combining circRNA Array and circBase, *IPO11* gene transcribed nine circRNA transcripts due to variable cyclization (Fig. S1E). Compared with other cycled transcripts, *circIPO11* transcript was abundant in tumors (Fig. S1F). In addition, *circIPO11* complementary upstream and downstream sequences were highly conserved in various species (Fig. S1G-J). Of note, *circIPO11* was more substantially expressed in tumor than peri-tumor tissues (Fig. 1D), oncospheres than non-sphere cells (Fig. 1E), and CSCs than non-CSCs (Fig. 1F). These results were further validated by Northern blot. In addition, we found that copy numbers of *circIPO11* in liver oncospheres and CSCs were about 200 per cell (Fig. 1G). Moreover, *circIPO11* was mainly localized in the nucleus via in situ hybridization of HCC samples (Fig. 1H), nuclear-cytoplasmic separation assay (Fig. 1I) and Immunofluorescence staining of human tumor tissues (Fig. 1J). Altogether, *circIPO11*, a conserved circRNA, is highly expressed in HCC tumors and liver CSCs that participates in the maintenance of liver CSC self-renewal.

### *CircIPO11* depletion impairs liver CSC self-renewal maintenance and inhibits HCC propagation

**Fig. 2:**
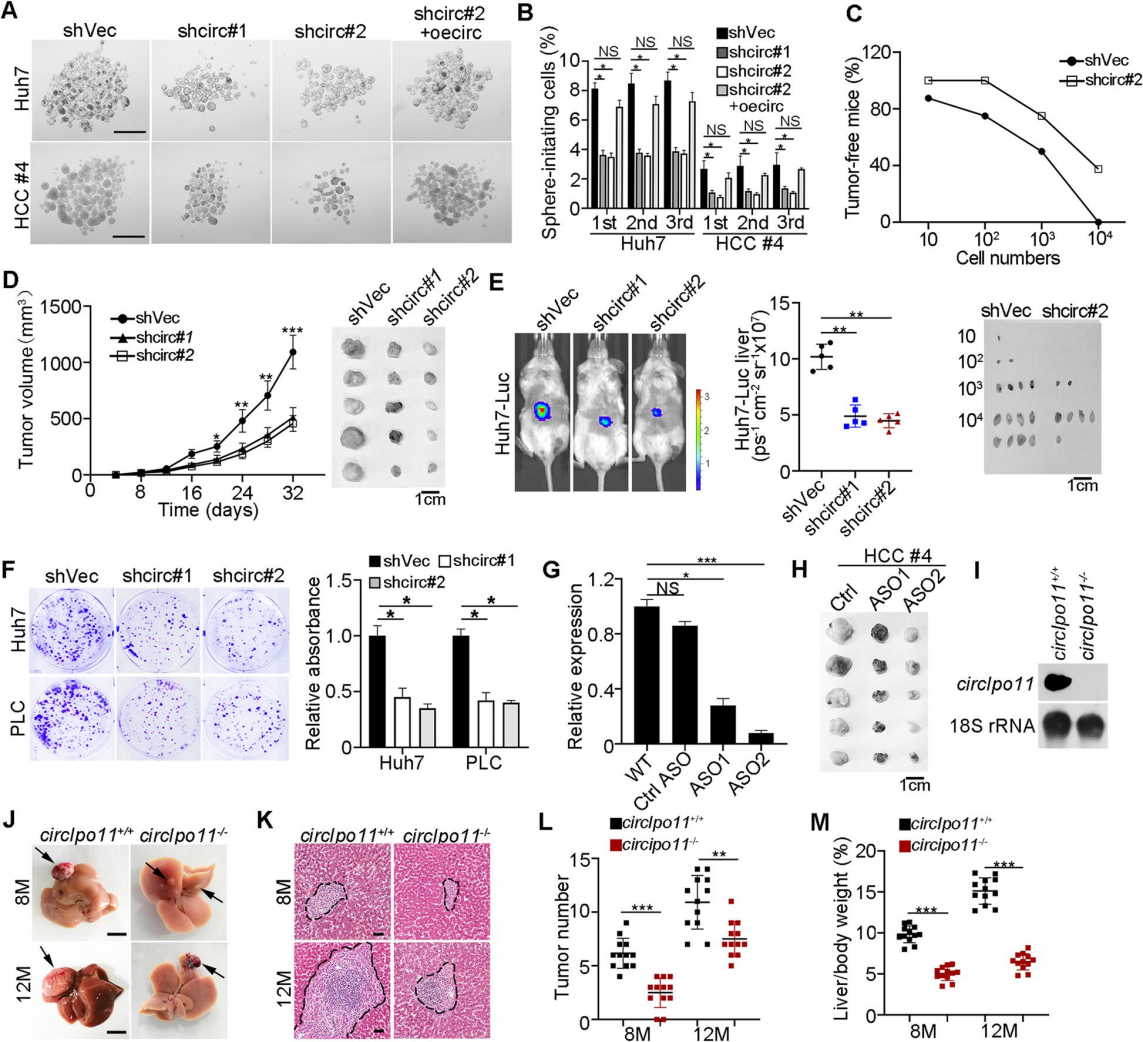
*CircIPO11* depletion impairs liver CSC self-renewal maintenance and inhibits HCC propagation. **A, B** *CircIPO11* depletion decreased the capacity of oncosphere formation in HCC cells. Overexpression of *circIPO11* (oecirc) rescued the sphere formation reduced by *circIPO11* depletion (**A**). Scale bar, 500 μm. statistical results of sphere-formation ratios were tested by serial sphere formation assays (**B**). Data are shown as means ± SD. **C** Limited-diluted *circIPO11* depletion or control Huh7 CSCs were subcutaneously injected into BALB/c nude mice for 3 months to detect tumor-bearing rates of mice. Tumor-free mice are counted (upper panel) and representative tumor images are shown (lower panel). *n* = 8 for each group. **D** 1 × 10^6^ *circIPO11*-depleted or control HCC cells were subcutaneously injected into BALB/c nude mice. Two effective shRNAs were used. Representative tumor images are shown (right panel). Results are shown as means ± SD. *n* = 5 for each group. **E** Orthotopic liver tumor imaging of Huh7-Luc cells transduced with control or shcirc#1 or #2 vectors. Representative images are shown (left panel), and statistical results are shown as means ± SD (right panel). *n* = 5 for each group. **F** Representative images of clone formation capacity in *circIPO11* depletion and control HCC cell lines (left panel), and statistical results are shown as means ± SD (right panel). **G** *CircIPO11* expression levels in ASO-treated (40 ng/ml) Huh7 cells were detected followed by qRT-PCR. ASOs (#1, #2 and #3) were designed and two effective ASOs (#1 and #2) for corresponding experiments. ASOs were listed in Table S1. **H** ASOs (#1 and #2) against *circIPO11* were used to treat HCC primary tumors in BALB/c nude mice. Twenty-five milligram per kilogram ASOs were injected around the tumor every 2 days (*n* = 5 mice per group). Scale bar, 1 cm. **I** *CircIpo11* was completely abolished in *circIpo11* KO mice by Northern blots. **J** Macroscopic tumor images of *circIpo11*^*+/+*^ and *circIpo11*^*−/−*^ livers after treated by DEN for 8 months (8 M) and 12 months (12 M). Black arrows indicate liver tumors. *n* = 12 for each group. Scale bar, 1 cm. **K** Representative HE images of *circIpo11*^*+/+*^ and *circIpo11*^*−/−*^ livers sections at 8 M and 12 M after DEN treatment. Scale bar, 100 μm. **L, M** Numbers of macroscopic tumors on liver surface (**L**), and liver-to-body weight ratios (**M**) were presented at 8 M and 12 M after DEN treatment. *n* = 12 for each group. *P < 0.05; **P < 0.01; *** *P* < 0.001 by two-tailed Student’s t-test. Data are representative of at least three independent experiments

In order to further explore the role of *circIPO11* in liver CSCs, we targeted the head-to-tail junction by lentivirus-mediated short hairpin RNA (shRNA) to obtain *circIPO11* depleted HCC cells and confirmed by qRT-PCR (Fig. S2A). Of note, *circIPO11* depletion did not affect intracellular levels of its parental gene *IPO11* (Fig. S2B, C). We observed that *circIPO11* depletion significantly reduced primary, secondary, and tertiary sphere formation capacity. In addition, overexpression of *circIPO11* could rescue the number of oncospheres (Fig. 2A, B). We found that the proportion of CD13^+^CD133^+^ cells decreased significantly when *circIPO11* was depleted (Fig. S2D, E).

We next wanted to determine the function of *circIPO11* in vivo. Extremely limiting dilutions of different cancer cell populations, and then transplants serial tumor into immunodeficient mice to measure their capacity to reform secondary tumors is the gold standard to assess CSC potential [29]. Through limiting dilution xenograft analysis, *circIPO11* depletion remarkably impaired CSC self-renewal and reduced tumor initiation (Fig. 2C). By limiting dilution analysis, *circIPO11* knockdown significantly reduced self-renewal capacity of liver CSCs (Fig. S2F). In addition, we subcutaneously injected 1 × 10^6^ *circIPO11* depletion or control cells into BALB/c nude mice. *CircIPO11* depletion cells remarkably reduced tumor propagation (Fig. 2D). To assess the role of *circIPO11* on orthotopic liver tumor development, *circIPO11* depletion and control cells were transfected with plasmids containing luciferase (Luc). We observed that *circIPO11* depletion dramatically reduced tumor growth in situ (Fig. 2E). To further determine the role of *circIPO11* in liver CSCs, we isolated liver CSCs (CD13^+^CD133^+^) and non-CSCs (CD13^−^CD133^−^) from Huh7-Luc cells, followed by depletion of *circIPO11* and transplantation into livers of immunodeficient mice. We observed that *circIPO11* knockdown dramatically suppressed xenograft tumor growth in CSCs but not in non-CSCs (Fig. S2G). Inhibition of *circIPO11* displayed poor proliferation capacity via clone formation assay (Fig. 2F). Then, we used two effective antisense oligonucleotides (ASOs) against *circIPO11* junction to investigate its therapeutic roles. We found ASOs treatment of HCC cell line could significantly inhibit *circIPO11* expression (Fig. 2G). We observed that the two ASOs significantly inhibited tumor growth in BALB/c nude mice (Fig. 2H). These data indicate that *circIPO11* promotes the self-renewal maintenance of liver CSCs.

Given that *circIPO11* was highly homologous in humans and mice, mouse ortholog *circIpo11* also has two exons originated from its parental gene *Ipo11* gene on chr 13 (Fig. S3A). Mouse c*ircIpo11* was further validated by sequencing (Fig. S3B). Since intronic complementary sequences flanking exons are necessary for formation of exonic circRNAs, the exonic circRNAs do not produce if either of intronic complementary sequences is missing [30]. Moreover, intronic complementary sequences verified by mini-gene assay played an important role in formation of *circIpo11* (Fig. S3C, D). Based on this, we next generated *circIpo11* knockout (KO) mice by deleting the upstream reverse complementary sequence via a CRISPR/Cas9 technology (Fig. S3E). *CircIpo11* was completely deleted in mice by northern bolt (Fig. 2I) and the upstream complementary pairing region was knocked out and confirmed by PCR (Fig. S3F). Meanwhile, the inability of the divergent primer to amplify the *circIpo11* fragment further indicated that *circIpo11* was successfully KO in mice by PCR (Fig. S3G). Similarly, *circIpo11* KO did not affect the expression of *Ipo11* gene (Fig. S3H). Of note, *circIpo11* KO mice had no apparent phenotypes, and displayed normal liver development. Diethylnitrosamine (DEN) has been commonly used to chemically induced HCC. We injected mice intraperitoneally injected with DEN. At 8th and 12th month after DEN treatment, sacrificed mice for macroscopic examination showed that *circIpo11*^−/−^ liver tumor foci were dramatically reduced in size compared with *circIpo11*^*+/+*^ littermate control mice (Fig. 2J). In addition, livers from DEN-treated *circIpo11*^−/−^ mice displayed much smaller and fewer nodular hyperplastic areas in histological sections than those of DEN-treated WT mice (Fig. 2K-L). The proportion of liver weight vs. body weight is another important factor that reflects tumor malignance [31]. Ratios of liver weights vs. body weights of *circIpo11*^−/−^ mice were much lower than WT mice (Fig. 2M). Collectively, *circIpo11* KO impairs the self-renewal maintenance of liver CSCs and suppresses tumor propagation as well.

### Overexpression of *circIPO11* promotes liver CSC self-renewal and tumorigenic capacity

**Fig. 3:**
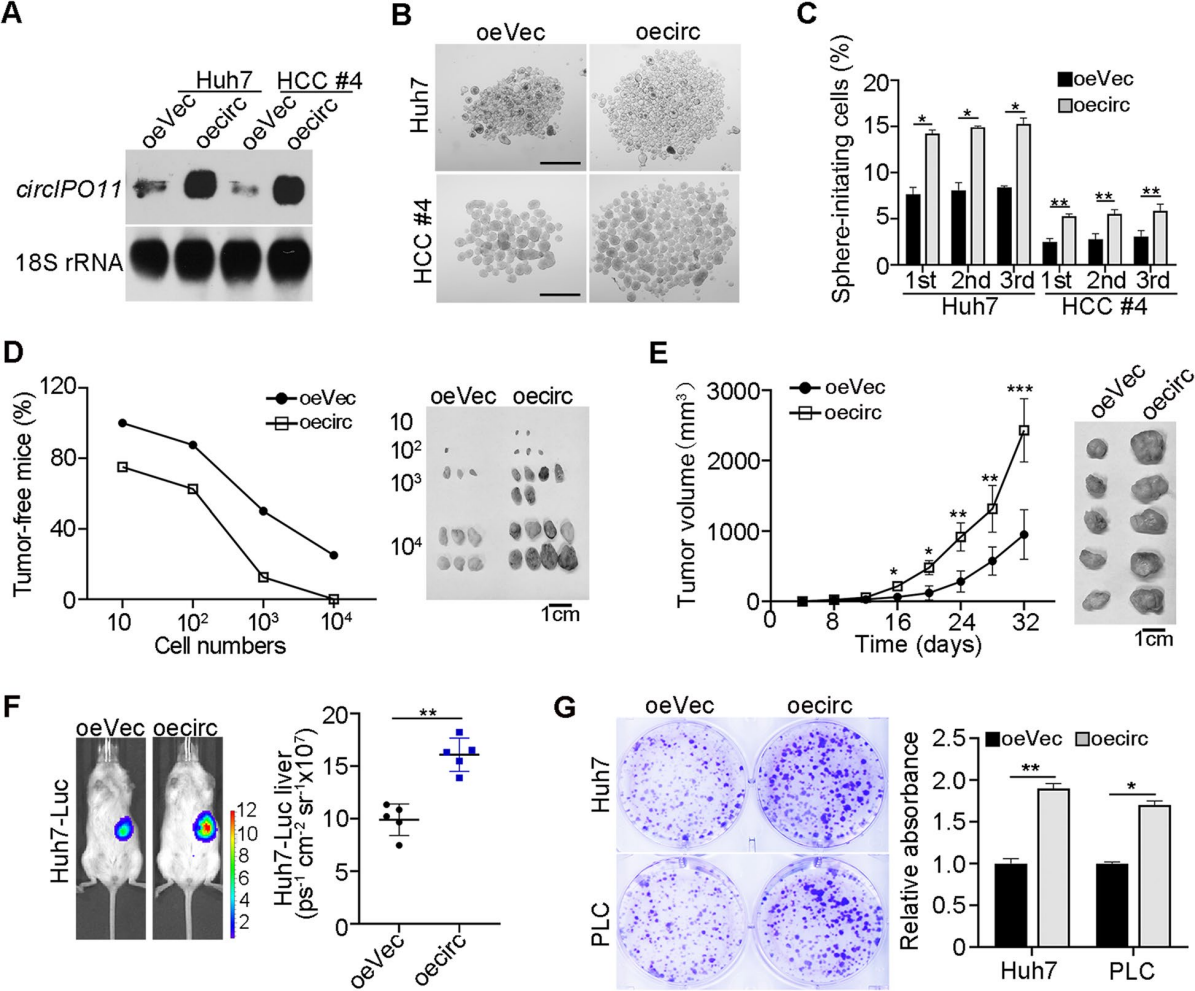
*circIPO11* overexpression promotes liver CSCs self-renewal and tumorigenic capacity. **A** *CircIPO11* overexpressed HCC cell lines and HCC primary cells were established with lentivirus carrying indicated mini-gene constructs, followed by Northern blot. oeVec, overexpression empty vector; oecirc, overexpression of *circIPO11*. *CircIPO11* and 18S rRNA (loading control) probes were used for Northern blot. Data are shown as means ± SD. **B, C** *CircIPO11* overexpression enhanced the capacity of oncosphere formation. Representative images (**B**) and statistical results of sphere-formation ratios (**C**) are shown. Scale bar, 500 μm. Data are shown as means ± SD. **D** Diluted *circIPO11* overexpression or control Huh7 CSCs were subcutaneously injected into BALB/c nude mice for 3 months to determine tumor-bearing rates of mice. Tumor-free mice are counted (left panel) and representative tumor images are shown (right panel). *n* = 8 for each group. **E** 1 × 10^6^ *circIPO11*-overexpressing or control HCC cells were subcutaneously injected into BALB/c nude mice. Results are shown as means ± SD. Representative tumor images are shown (right panel). *n* = 5 for each group. **F** Orthotopic liver tumor imaging of Huh7-Luc cells transduced with control or oe*circIPO11* vectors. Representative pictures are shown (left panel), and statistical results are shown as means ± SD (right panel). *n* = 5 for each group. **G** Overexpression of *circIPO11* enhanced the capacity of clone formation. Representative images and statistical results are shown. *P < 0.05; **P < 0.01; *** *P* < 0.001 by two-tailed Student’s t test. Data are representative of at least three independent experiments

We next overexpressed *circIPO11* in HCC cells (Fig. 3A, Fig. S2H). Of note, *circIPO11* overexpression did not affect the expression level of its parental gene *IPO11* (Fig. S2I, J). *CircIPO11* overexpression dramatically increased the capacity of oncosphere formation (Fig. 3B). Serial passage assays illustrated that *circIPO11* overexpression improved the capacity of liver CSC self-renewal (Fig. 3C). Though limiting dilution, subcutaneous tumor formation, and in vivo imaging assay, we found that *circIPO11* overexpression could significantly increase the proportion of tumor-bearing mice (Fig. 3D) and promote HCC progression of in situ (Fig. 3E, F). Meanwhile, *circIPO11* overexpression significantly increased self-renewal capacity of CSCs by limiting dilution analysis (Fig. S2K). In parallel, *circIPO11* overexpression enhanced proliferation of HCC cells (Fig. 3G). Taken together, *circIPO11* overexpression promotes self-renewal and tumorigenic capacity of liver CSCs.

### *CircIPO11* associates with TOP1 in liver CSCs

**Fig. 4:**
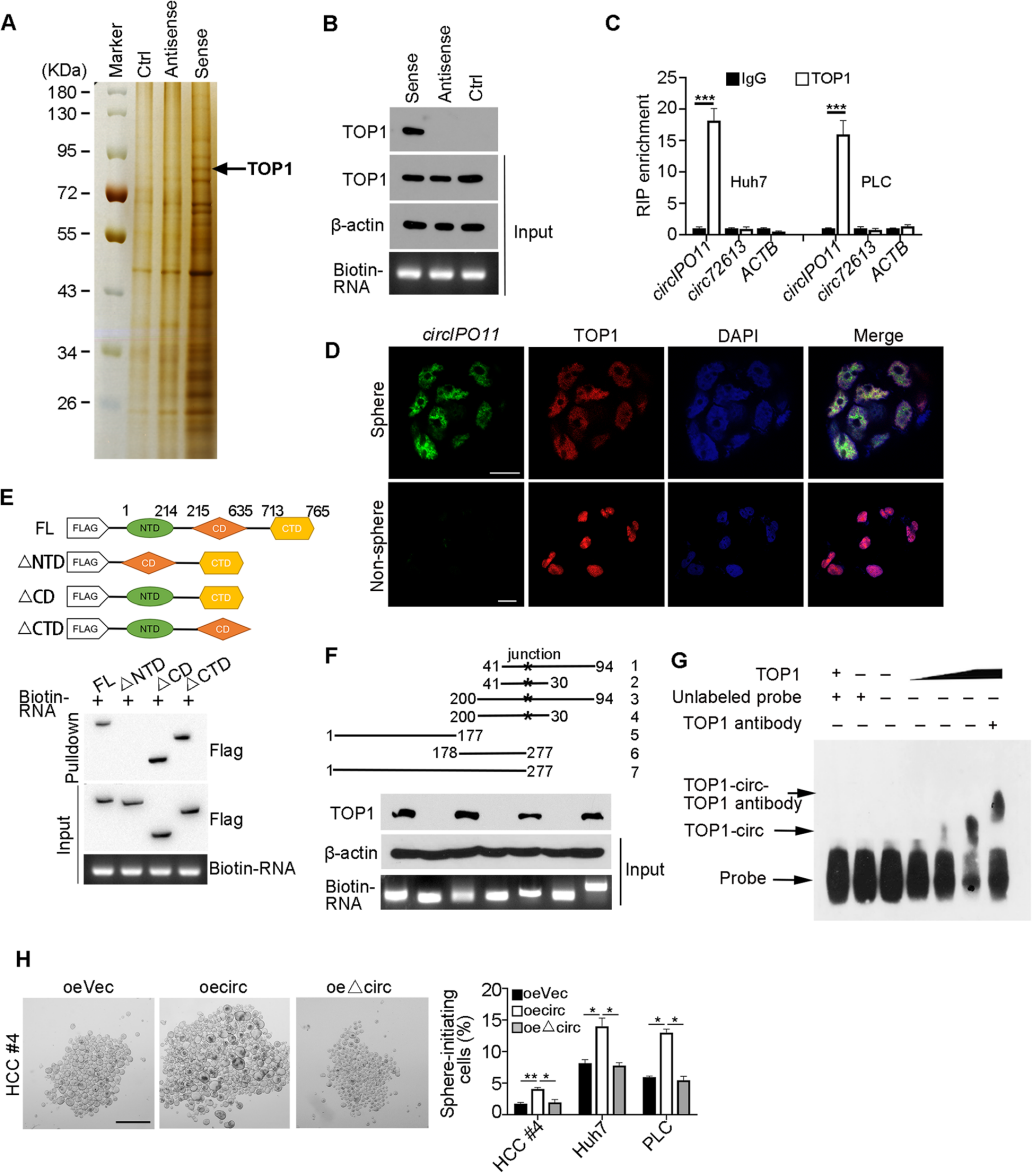
*CircIPO11* associates with TOP1 in liver CSCs. **A** RNA pulldown assay was performed in oncosphere cell lysates using biotinylated *circIPO11* junction sequences (sense), antisense and IPO11 intron sequences (Ctrl), followed by mass spectrometry. Differential band to bind *circIPO11* was identified as TOP1 (black arrow). **B** Interaction of *circIPO11* with TOP1 was confirmed from oncospheres derived from HCC samples. β-actin was used as a loading control. **C** Huh7 and PLC oncospheres were used for RIP assay, and followed by qRT-PCR. *circRNA72613* and *ACTB* were used as controls. Results are shown as means ± SD. **D** *CircIPO11* was annotated by RNA FISH, followed by immunofluorescence staining of TOP1 in oncosphere and non-sphere cells. Scale bar, 30 μm. **E** Domain mapping analysis of *circIPO11*-binding domains of TOP1 protein. Different domains of TOP1 protein were incubated with *circIPO11*, followed by RNA pulldown assay and Western blot. NTD, N-terminal domain; CD, core domain; CTD, C-terminal domain. **F** Truncated fragments of biotinylated *circIPO11* were incubated with HCC oncosphere lysates, followed by RNA pulldown assay and Western blot (lower panel). Schematic diagram of *circIPO11* truncated fragments (upper panel). **G** Biotin-labeled *circIPO11* (nt 31–94) RNA probe was incubated with TOP1 for RNA EMSA. **H** *CircIPO11* and Δ*circIPO11* were overexpressed in HCC primary cells and HCC cell lines, followed by oncosphere formation assay. Representative images (left panel) and statistical results are shown (right panel). Scale bar, 500 μm. *P < 0.05; **P < 0.01; *** *P* < 0.001 by two-tailed Student’s t test. Data are representative of at least three independent experiments

We then investigated the molecular mechanism by which *circIPO11* regulated liver CSCs. Through circBank and NCBI website, none of the open reading frame (ORF) was potentially existed, suggesting that *circIPO11* is a noncoding RNA. Moreover, there was also little miRNA binding sites in *circIPO11*, indicating that *circIPO11* does not serve as microRNA sponges. In addition, there was no change of its parental gene *IPO11* by knockdown or overexpressing *circIPO11* (Fig. S2B, C, Fig. S2G, H). Interestingly, based on circBank, *circIPO11* was predicted to have protein-binding potential. On the other hand, *circIPO11* harbored some stem-loop structures through prediction analysis by the RNAfold Web Services (Fig. S4A), suggesting that *circIPO11* may associate with proteins to perform its function. RNA pull down assay using biotin-labeled probes was performed for searching potential associated proteins of *circIPO11* in liver oncospheres. After mass spectrometry analysis, we identified topoisomerases 1 (TOP1), relaxing supercoiled DNA during gene replication and transcription, as a candidate binding protein (Fig. 4A and Fig. S4B). The interaction of *circIPO11* with TOP1 was further validated by Western blot (Fig. 4B). In addition, we evaluated whether *circIPO11* interacted with TOP1 by structure prediction. *CircIPO11* secondary structure was submitted to RNA Composer to generate the 3D structure (PDB: 1a36). NPDock was then used to calculate the molecular docking between *circIPO11* and TOP1, which indicated that *circIPO11* perfectly docked TOP1 (Fig. S4A). Through RNA immunoprecipitation (RIP) assay, TOP1 was able to precipitate *circIPO11* in oncosphere lysates, rather than other variable cyclizations derived from *IPO11* (Fig. 4C). Co-localization of *circIPO11* with TOP1 was further confirmed by immunofluorescence staining in oncosphere cells (Fig. 4D). And we found they were mainly located in the nucleus. The NTD domain of TOP1 protein was necessary to bind *circIPO11* via domain mapping assays (Fig. 4E), suggesting *circIPO11* directly binds to TOP1. In addition, the segment 31–94 nt of *circIPO11* was sufficient to bind TOP1 through fragment mapping (Fig. 4F). Finally, the connection of *circIPO11* segment (31–94 nt) with TOP1 was further verified by RNA electrical mobility shift assay (EMSA) (Fig. 4G).

We next overexpressed *circIPO11* lacking the TOP1-binding region (Δ*circIPO11*) in HCC cells. The capacity of oncosphere formation (Fig. 4H) and cell proliferation (Fig. S4C) was almost abrogated in Δ*circIPO11* overexpressed HCC cells, suggesting that the interaction of *circIPO11* with TOP1 is necessary for the self-renewal of liver CSCs. Collectively, we conclude that *circIPO11* interacts with TOP1 in liver CSCs.

### *CircIPO11* recruits TOP1 onto GLI1 promoter to activate its expression

**Fig. 5:**
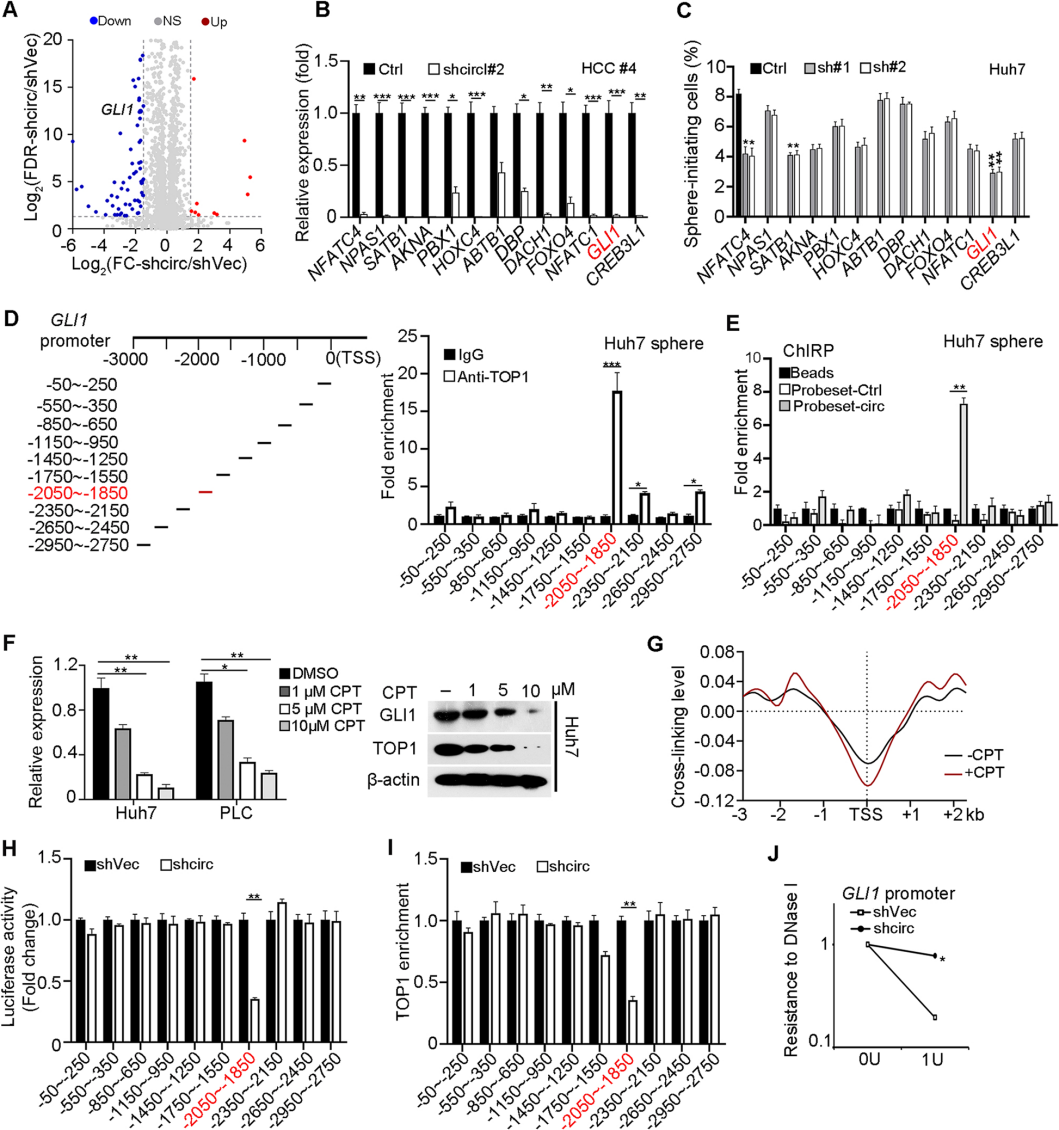
*CircIPO11* recruits TOP1 onto GLI1 promoter to activate its expression. **A** Volcano plot of differentially expressed transcription factors in *circIPO11*-depleted and control liver CSC cells. **B** Expression levels (normalized to ACTB) of top 13 downregulated transcription factors in HCC primary cells were detected by qRT-PCR, and shVec as Ctrl for each gene. Results are shown as means ± SD. **C** Each of the top 13 downregulated transcription factors was silenced in HCC cell lines by shRNA, followed by oncosphere formation assay (*n* = 3). **D** ChIP was performed to identify binding regions of TOP1 on *GLI1* promoter in HCC oncospheres, followed by qPCR. IgG enrichment served as a control. Results are shown as means ± SD. **E** CHRIP was performed to identify GLI1 promoter enrichment of *circIPO11* in HCC oncospheres (*n* = 3). **F** Expression of GLI1 in HCC cells treated with different concentrations of CPT, followed by qRT-PCR and Western blot. Results are shown as means ± SD. **G** Psoralen photobinding assay was performed to detect superhelix structure on *GLI1* promoter. **H** Luciferase reporter assays were performed in *circIPO11*-depletion and control cells. Data are shown as means ± SD. **I** ChIP-qPCR analysis of TOP1 enrichment on *GLI1* promoter in *circIPO11*-depletion and control cells. Results are shown as means ± SD. **J** *CircIPO11*-depletion reduced chromatin accessibility at the GLI1 promoter by DNase I digestion assays. Results are shown as means ± SD. *P < 0.05; **P < 0.01; *** *P* < 0.001 by two-tailed Student’s t test. Data are representative of at least three independent experiments

To further identify target genes of *circIPO11*, we performed transcriptome microarray analysis using *circIPO11* depleted and control oncosphere cells (Fig. 5A, Fig. S5A). Top 13 downregulated transcription factors (TFs) were selected to further verify their expression in *circIPO11* depleted oncosphere cells by qRT-PCR (Fig. 5B). We then obtained these 13 TFs silenced HCC cell lines via shRNAs and performed sphere formation assays. Among them, knockdown of GLI1 dramatically affected sphere formation ability in Huh7 cell lines (Fig. 5C). GLI1 (GLI family zinc finger 1), acts exclusively as an activator of Hh signaling, since it lacks a repressor domain compared to GLI2 and GLI3 [32]. The strength and downstream gene levels of Hh pathway are regulated by the ratio of GLI1 activators and inhibitors forms. Hh/GLI signaling mediates various cellular activities by activating distinct target gene sets, especially in the field of stem cells [33], including hair follicle stem cells, cancer stem cells, and follicle stem cell. We then performed chromatin immunoprecipitation (ChIP) assays in HCC oncospheres with anti-TOP1 antibody. We analyzed a 3 kb region upstream of the *GLI1* gene transcription start site (TSS). We found that TOP1 was enriched at the − 2050 to − 1850 bp region of the *GLI1* promoter (Fig. 5D). In addition, dual luciferase reporter assay also revealed TOP1 bound to the same locus of *GLI1* promoter. (Figs. S5B, C).

To further explore the role of *circIPO11* in regulating GLI1 expression, we performed ChIRP-PCR assay. We found that *circIPO11* transcripts were mainly distributed on the − 2050 to − 1850 bp region of the *GLI1* promoter as same as TOP1 did (Fig. 5E). Moreover, dual luciferase reporter assays further verified *circIPO11* depletion dramatically decreased luciferase activity of the − 2050 to − 1850 bp region, illustrating *circIPO11* could recruit TOP1 to *GLI1* promoter (Fig. 5H). Furthermore, *circIPO11* depletion significantly reduced TOP1 enrichment on the *GLI1* promoter (Fig. 5I). In addition, *circIPO11* overexpression remarkedly increased TOP1 enrichment on the *GLI1* promoter (Fig. S4D). Camptothecin (CPT), a classic inhibitor of TOP1 [34], was used to treat cells with different doses and detected the expression of GLI1 by qRT-PCR and Western blot. We found that when TOP1 was inhibited by 10 μM CPT, and the intracellular level of GLI1 was completely repressed (Fig. 5F). Similarly, through psoralen photobinding assay [35], we observed that there was a negative superhelix structure on the region of GLI1 promoter -2 kb (Fig. 5G). While *circIPO11* and TOP1 bound to this region, the negative superhelix structure would be released to initiate the transcription of GLI1. We next tested chromatin accessibility of *GLI1* promoter through DNase I sensibility assay. We found that *circIPO11* depletion dramatically wrinkled chromatin accessibility of the *GLI1* promoter (Fig. 5J). In contrast, *circIPO11* overexpression substantially opened the chromatin accessibility of the *GLI1* promoter (Fig. S5E). Next, we constructed TOP1 deleted HCC cell lines by CRISPR/Cas9 technology and identified them by Western blot (Fig. S5F). And then dual luciferase reporter assays further verified TOP1 depletion dramatically decreased the luciferase activity of the − 2050 to − 1850 bp region (Fig. S5G). In general, these data suggest that *circIPO11* recruits TOP1 to the *GLI1* promoter to initiate its expression.

### GLI1 initiates Hh signaling activation for liver CSC self-renewal

**Fig. 6:**
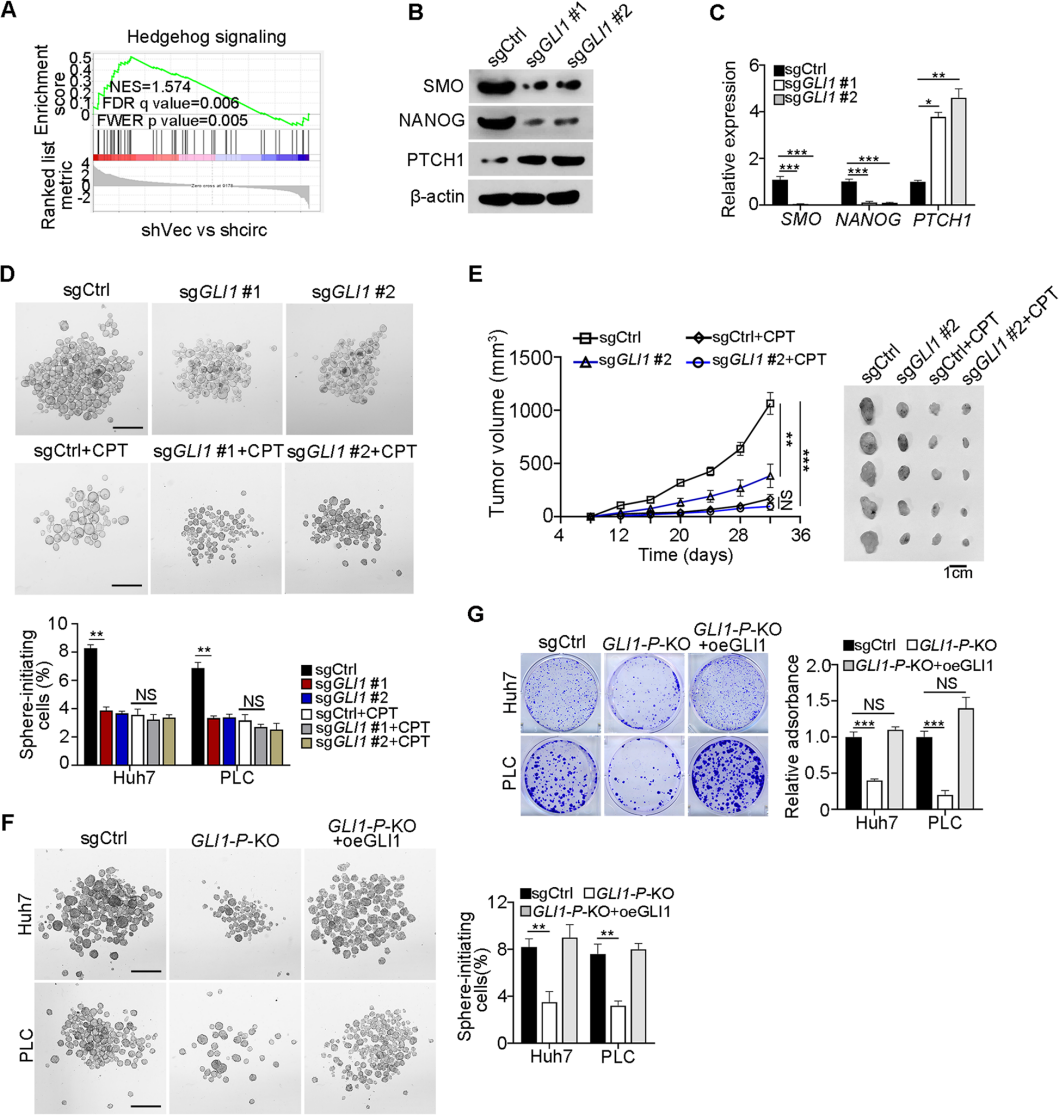
GLI1 promotes Hh signaling activation to drive liver CSC self-renewal. **A** GSEA indicated significantly altered genes were enriched in Hh signaling in *circIPO11* depleted liver CSC cells. NES, normalized enrichment score; FDR, false discovery rate; FWER, familywise error rate. **B** GLI1-deleted HCC primary cells were generated by CRISPR/Cas9 approach. Whole cell lysates of GLI1 KO cells were examined with SMO, Nanog and PTCH1 antibody. **C** Hh signaling target genes and downstream stemness genes were tested in GLI1 KO cells by qRT-PCR. Data are shown as means ± SD. **D** Oncosphere formation assay was performed by GLI1-deleted HCC cell lines. Representative images (upper panel) and statistical results (lower panel) are shown. Scale bar, 500 μm (*n* = 3 cell cultures). **E** 1 × 10^6^ GLI1-deleted cells, GLI1-deleted cells with CPT, control cells and control cells with CPT were subcutaneously injected into BALB/c nude mice. Tumor-volume curves (left panel) and representative tumor images are shown (right panel). Data are shown as means ± SD (*n* = 5 mice). **F** GLI1 overexpression rescued the reduced sphere formation capacity caused by *GLI1* promoter deletion. *GLI1-P*-KO, the binding region of TOP1 to *GLI1* promoter knockout; Results are shown as means ± SD. Scale bar, 500 μm. **G** GLI1 overexpression rescued the reduced clone formation capacity caused by *GLI1* promoter deletion. *GLI1-P*-KO, the binding region of TOP1 to the *GLI1* promoter knockout; Results are shown as means ± SD. **P < 0.01; *** *P* < 0.001 by two-tailed Student’s t test. Data are representative of at least three independent experiments

To further test the downstream signaling pathway of *circIPO11* in liver CSCs, integrative gene set enrichment analysis (GSEA) of transcriptome microarray data were conducted. We noticed that the target genes of Hh signaling were remarkably suppressed with *circIPO11* depletion, suggesting Hh signaling was involved in the regulation of liver CSCs (Fig. 6A). We recently showed that Hh signaling pathway plays an important role in the regulation of liver CSCs [12]. We then constructed *GLI1* knockout HCC cells using CRISPR/Cas9 technology and identified them by sequencing (Fig. S6A, B). GLI1 protein was completely abrogated by Western blot detection (Fig. S6C). Of note, GLI1 deletion dramatically changed the expression levels of Hh signaling target genes and downstream stemness gene (Fig. 6B, C). As expected, GLI1 KO cells could reduce oncosphere formation (Fig. 6D) and cell proliferation capacity (Fig. S6D). Since CPT is a classical inhibitor against TOP1, we then used CPT to treat GLI1 KO cells for oncosphere formation assays. We noticed that treatment of CPT could not impair GLI1 deletion-induced oncosphere formation in vitro (Fig. 6D). Unexpectedly, we found an interesting phenomenon that tumors were significantly smaller through subcutaneous tumor formation experiment after treatment with CPT (Fig. 6E). We speculated that the inhibition of TOP1 by CPT might also have damage on normal cells, thus causing the storm of immune factors and increasing the killing effect on tumors, finally leading to the reduction of tumors in vivo*.* To further examine the role of *GLI1* in liver CSCs, we isolated liver CSCs and non-CSCs from Huh7 cells, followed by depletion of *GLI1* and subcutaneous transplantation into BALB/c nude mice. We found that *GLI1* KO dramatically inhibited xenograft tumor growth in CSCs (Fig. S6E). In addition, we noticed that proliferation-related transcription factors and stemness factors were significantly upregulated in *circIPO11* overexpressed tumor cells (Fig. S6F). Finally, we depleted GLI1 in HCC cells with versus without *circIPO11* overexpression, followed oncosphere formation assays. We found that GLI1 depletion in *circIPO11* overexpressed HCC cells could not promote oncosphere formation capacity compared with control HCC cells.

Furthermore, the TOP1 binding region of *GLI1* promoter (*GLI1-P*-KO) was deleted in HCC cells using CRISPER/Cas9 system (Fig. S6G). We observed that *GLI1-P*-KO decreased sphere formation and cell colony formation, whereas GLI1 overexpression rescued this phenomenon (Fig. 6F, G). However, *circIPO11* overexpression did not improve sphere formation in *GLI1-P*-KO cells (Fig. S6H), indicating the TOP1 binding region of *GLI1* promoter was required for *circIPO11* function. Taken together, *circIPO11*-mediated GLI1 upregulation enhances Hh signaling activation that initiates the self-renewal maintenance of liver CSCs and tumor propagation.

### GLI1 and TOP1 expression are positively related to HCC severity

**Fig. 7:**
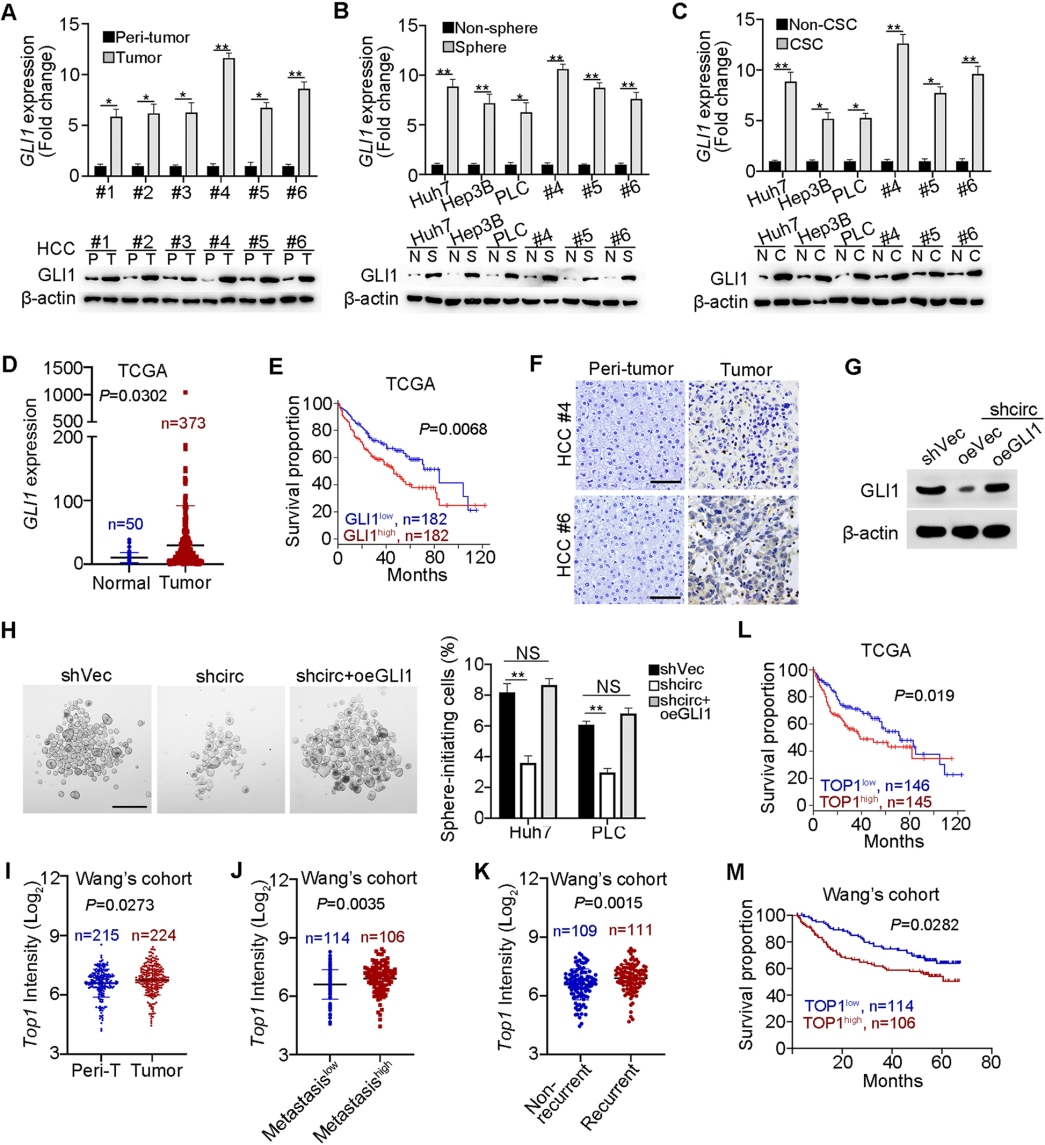
GLI1 and TOP1 are positively correlated with HCC severity. **A-C** GLI1 was examined in HCC tumor and peri-tumor tissues (**A**), oncospheres and non-sphere cells (**B**), and liver CSCs and non-CSCs (**C**). **D, E** Expression levels of GLI1 in HCC samples (**D**), Kaplan-Meier survival analysis (**E**), provided by TCGA. **F** Immunohistochemistry with anti-GLI1 was performed in HCC samples. Scale bars, 50 μm. **G** GLI1 was overexpressed in *circIPO11*-depleted cells. β-actin served as a loading control. **H** GLI1 overexpression restored the reduced sphere formation capacity caused by *circIPO11* deletion. Scale bars, 500 μm. **I-K** Expression levels of TOP1 in HCC samples (**I**), HCC metastatic patients (**J**) and recurrent patients (**K**) provided by Wang’s cohort (GSE14520). **L, M** Kaplan–Meier survival curves of HCC samples from TCGA (**L**) and Wang’s cohort (**M**). Based on the TOP1 expression levels, patients were divided into two groups. *P < 0.05; **P < 0.01; by two-tailed Student’s t test. Data are representative of at least three independent experiments

To further examine the function of GLI1 in liver CSCs, we detected the expression levels of GLI1 in HCC tumors and peri-tumor tissues, oncespheres and non-sphere cells, as well as liver CSCs and non-CSCs. We found that GLI1 was highly expressed in tumor tissues, liver CSCs and oncosphere cells (Fig. 7A-C). High expression of GLI1 in HCC samples was further validated by TCGA database (Fig. 7D). The expression of GLI1 was positively correlated with the poor prognosis of HCC patients (Fig. 7E). Of note, GLI1, mainly localized in the nucleus, was highly expressed in HCC samples via in situ hybridization (Fig. 7F). These data suggest that GLI1 expression levels are positively correlated with HCC severity. *CircIPO11* depletion decreased the expression of GLI1 (Fig. 7G). Moreover, GLI1 overexpression in *circIPO11* depleted cells remarkably enhanced GLI1 expression and sphere formation (Fig. 7G, H). To further determine the correlation of *circIPO11*, TOP1 and GLI1, we examined their expression levels among 29 HCC samples. We observed that *circIPO11* expression levels were positively related to expression levels of GLI1 and TOP1 (Fig. S7A, B). Moreover, GLI1 expression levels were positively correlated with TOP1 expression levels (Fig. S7C). In addition, *circIPO11* knockdown and GLI1 KO dramatically suppressed proliferation of CSCs, but not affected cell death (Fig. S7D-G).

We further analyzed the expression of TOP1 with Wang’s cohort (GSE14520). We noticed that TOP1 was also highly expressed in HCC tumor tissues (Fig. 7I). In addition, the expression of TOP1 was significantly correlated with HCC patients’ survival, metastasis and recurrence (Fig. 7J-M), indicating that TOP1 expression levels are related to the severity of HCC. Taken together, GLI1 and TOP1 expression are positively correlated with HCC severity.

## Discussion

Cancer stem cells (CSCs) have been considered to be the source of tumor initiation, drug resistance, metastasis, and recurrence [36]. Some circRNAs play critical roles in many cellular processes, particularly in cancer. circGprc5a, is highly expressed in bladder CSCs and regulates the self-renewal of CSCs through a coding peptide. However, how circRNAs regulate liver CSC stemness remains enclusive. Here we identified a conserved circRNA named *circIPO11* that is significantly increased in liver CSCs and necessary for the self-renewal maintenance of liver CSCs. Mechanistically, *circIPO11* recruits TOP1 onto *GLI1* promoter to trigger its expression, leading to activation of Hh signaling. Moreover, GLI1 is also highly expressed in liver CSCs. TOP1 expression is positively correlated with the survival, metastasis and recurrence of HCC patients. Targeting *circIPO11* and TOP1 has synergistic antitumor effects on HCC patient-derived tumor cells (PDC) models.

Compared with traditional linear RNAs, circRNAs are more stable and hard to degrade owing to their covalently closed circular structure, which is not affected by RNA exonuclease [37]. Because circRNAs have a very long half-life and tissue-specific features, their functions are more complicated than expected [38]. Due to the moderate conservation of most circRNAs, it is difficult to use animal models to research their physiological roles and test their therapeutic effects under pathological effects. In this study, we therefore focus on identifying highly conserved circRNAs and exploring their roles in tumorigenesis. To identify evolutionarily conserved circRNAs, we innovatively performed homology screening from differential circRNAs compared with tumor and peri-tumor tissues. We found that *circIPO11* was highly conserved across various species. Its mouse ortholog is *circIpo11* from murine *Ipo11* gene including 2 exons. To explore the function of circRNA in vivo, we generated *circIpo11* knockout (KO) mice by deleting the upstream reverse complementary sequence using CRISPR/Cas9 technology. Intriguingly, *circIpo11*^*−/−*^ liver tumor foci were dramatically reduced in size than *circIpo11*^*+/+*^ littermates, suggesting that *circIPO11* play a critical role in tumorigenesis and progression of liver cancer. Up to date, the relationship between circRNA and its linear mRNA is still controversial. Some studies showed that circRNA competes with its parental RNA [39] and others have suggested that circRNAs can promote the transcription of their parental genes *in cis* [40]*.* In addition, some circRNAs, highly specific in brain, are independent of their linear isoforms generated from their parental genes [15, 41]. In this study, we showed that *circIPO11* expression is not significant correlation with its parental gene *IPO11* in liver CSCs, suggesting a critical role of *circIPO11* in the regulation of liver CSCs. *CircIPO11* is an exon circular RNA consisting of the fourth and fifth exons of *IPO11* gene. The function of IPO11 is controversial, which has been reported as a tumor suppressor [42]. However, this observation was most challenged by IPO11 as a required factor for β-catenin-mediated transcription in colorectal cancer [43]. We found that IPO11 expression is comparable between HCC tumor and peri-tumor tissues and has no significant correlation with HCC patients’ prognosis. Additionally, *circIPO11* depletion does not affect the expression of its parental gene *IPO11* and other IPO11-derived circRNAs. We thus conclude that *circIPO11* regulates the self-renewal of liver CSCs in *trans*.

Reorganization of chromatin and DNA topological pressure are intrinsic to transcription. Accumulated data on the dynamics of DNA and DNA-binding elements during transcription provide new insights into the mechanism of gene expression [44]. DNA topoisomerases induce either single or double stranded DNA breaks to regulate DNA topological status during replication, transcription, recombination, and chromatin remodeling [45]. On the one hand, TOP1 acts as an elongation factor by relaxing topological pressure in the process of transcription, such as transcription of the Hsp70 genes in Drosophila [46]. On the other hand, TOP1 initiates transcription by binding to the promoter of target gene that impacts its expression [47]. In addition, there exists a dynamic superhelix structure spread ~ 1.5 kilobases upstream of the transcription starting site of the target genes, as the binding site of TOP1 [35]. Recent studies have shown that inhibition of TOP1 in HCC can improve the sensitivity to chemical drugs [23, 48], but the relationship between TOP1 and liver CSCs is still unknown. Here we showed that *circIPO11* can interact with TOP1 and *GLI1* promoter to produce relaxing supercoiled DNA, leading to the transcription of *GLI1* gene.

The Hh signaling pathway is involved in numerous biological processes including embryonic development, tissue homeostasis, regeneration and healing. Aberrant Hh signaling may cause various human malignancies and CSCs [9, 49]. Hh pathway is inhibited by the unliganded form of receptor patched1 (PTCH1) in the absence of Hh ligands, including Sonic (Shh), Desert (Dhh), and Indian (Ihh). Following ligands engagement, PTCH1 promotes the activation and ciliary entry of the transmembrane protein Smoothened (Smo), which activates GLI transcription factors to initiate tumorigenesis. GLI1, as a downstream effector of Hh signaling, can promote the transcription of some crucial stemness genes such as NANOG. In addition, GLI1 expression is epigenetically regulated by chromatin remodeling complexes. Some studies have shown that ZNF521 can interact with the NuRD complex onto *GLI* promoter to activate its expression [50]. However, how Hh signaling is activated and regulates the progression of HCC is still rarely explored. Our findings reveal that Hh signaling mediated by activation of GLI1 plays a critical role in the self-renewal of liver CSCs and liver tumorigenesis.

Some studies have reported circRNAs as novel potential biomarkers for cancer. For example, exosomal *circNRIP1* serves as a biomarker for gastric cancer diagnosis [51]. Therapies targeting cancer-associated circRNAs are also under investigation. ASO therapy has been applied in neural diseases in non-human primates and human clinical trials through intrathecal administration, without serious adverse reactions [52]. ASO therapy has been shown to be safe in human clinical trials by intrathecal administration [53]. In this study, we revealed that ASOs against *circIPO11* can dramatically reduce tumor size, which indicates that targeting *circIPO11* by ASOs could be potential therapeutic strategy for HCC patients. However, the efficient delivery of these oligonucleotides to tumors and the eliciting of an immune response still require further investigation.

## Conclusion

In summary, our findings reveal that *circIPO11* initiates the self-renewal of liver CSCs and promotes the propagation of HCC. Mechanistically, we first discovered that *circIPO11* recruits TOP1 to GLI1 promoter to trigger its transcription, leading to the activation of Hedgehog signaling. Our findings provide a novel potential target for the treatment of HCC patients.

## Supplementary Information

**Additional file 1: Figure S1.** Characterization of upregulated *circIPO11* in HCC tumor tissues. (A) Validation of circRNAs by DNA sequencing. PCR products with divergent primers were sequenced. Divergent primers were listed in Table S3. (B) Using complementary DNA (cDNA) and genomic DNA (gDNA) as templates, *circIPO11* was amplified with divergent (grey arrowheads) and convergent primers (black arrowheads). *Gapdh* was used as a positive control. (C) Total RNAs extracted from HCC oncospheres were digested with or without 2 U/μg RNase R for 1 h at 37 °C, followed by qPCR analysis, *IPO11* and *ACTB* were used as positive controls. Data are shown as means ± SD. (D) HCC cell lines were treated with 2 μg/ml actinomycin D for 14 h, and whole RNAs were extracted for qPCR analysis. *IPO11* and *ACTB* were used as positive controls. Data are shown as means ± SD. (E) Schematic representation of nine circRNAs derived from *IPO11* transcript due to variable cyclizations. (F) Expression levels of the variable cycled circRNAs were examined by qRT-PCR. *CircIPO11* was the only circular RNA that highly expressed in tumor, oncospheres and liver CSCs. Data are shown as means ± SD. (G) Sequence conservation analysis of *circIPO11* in vertebrates from zebrafish to human. The scores listed in the table were identified by the WATER algorithm. (H, I) Conservation analysis of upstream (H) and downstream (I) complementary intron sequence of *circIPO11* in vertebrates. (J) Match information of complementary introns of *circIPO11* in vertebrates. U, upstream complementary intron sequences; D, downstream complementary intron sequences. **P < 0.01; *** *P* < 0.001 by two-tailed Student’s t test. Data are representative of at least three independent experiments. **Figure S2.** *CircIPO11* is required for the self-renewal of liver CSCs. (A) *CircIPO11* expression was detected by qRT-PCR in HCC cell lines and HCC primary tumor cells. Data are shown as means ± SD. (B, C) Expression levels of IPO11 in *circIPO11*-depleted HCC cells were detected by Western blot (B) and qRT-PCR (C). β-actin was used as a loading control. Data are shown as means ± SD. (D) CD13 and CD133 subpopulations in HCC primary tumor cells were detected by flow cytometry. (E) CD13^+^CD133^+^ subpopulations were sorted from *circIPO11*-depleted and control HCC cell lines, followed by flow cytometry analysis. (F) Tumorigenic cell frequencies in *circIPO11*-depleted and empty vector control (shVec) cells were analyzed with a limiting dilution assay (http://bioinf.wehi.edu.au/software/elda/). Data are shown as means and 95% confidence intervals (*n* = 8). (G) Orthotopic liver tumor imaging of CSCs and non-CSCs with and without *circIPO11* transduced with Luc vectors. Representative images are shown (left panel), and statistical results are shown as means ± SD (right panel). *n* = 3 for each group. (H) *CircIPO11* was overexpressed in HCC cell lines and HCC primary tumor cells. Data are shown as means ± SD. (I, J) Expression of *circIPO11* did not affect the expression of its parental gene *IPO11* by qRT-PCR (I) and Western blot (J). β-actin was used as a loading control. Data are shown as means ± SD. (K) Tumorigenic cell frequencies in *circIPO11*-overexpressing and control (oeVec) cells were determined with limiting dilution assay (http://bioinf.wehi.edu.au/software/elda/). Data are shown as means and 95% confidence intervals (*n* = 8). *** *P* < 0.001 by two-tailed Student’s t test. Data are representative of at least three independent experiments. **Figure S3.** Generation of *circIPO11* knockout mice. (A) Schematic representation of mouse *circIPO11*. Convergent primers for linear *Ipo11* were denoted in black arrowheads, and divergent primers agaisnt *circIPO11* were denoted in red arrowheads. E1, exon 1. (B) Validation of *circIPO11* by DNA sequencing in mice. PCR products with divergent primers were sequenced. (C, D) Schematic representation of mini-gene assay was shown (C). circ△up: missing upstream intronic complementary sequences flanking exon 4. circ△down: missing downstream intronic complementary sequences flanking exon 5. Identification of complementary intron sequences were required for *circIPO11* formation, followed by qRT-PCR (D). (E) Schematic representation for *circIpo11* KO mice. The upstream complementary sequences (#1) were deleted using CRISPR/Cas9 system. Corresponding sgRNAs were shown in Table S2. (F) *CircIpo11*^−/−^ mice were verified by agarose gel electrophoresis. Genome DNA of *circIpo11*^*+/+*^ and *circIpo11*^−/−^ mice was used for PCR template. WT allele had a PCR length of about 222 bp and deficient allele had a PCR length of about 336 bp. (G) Using complementary DNA (cDNA) and genomic DNA (gDNA) as templates, *circIpo11* was detected in WT or *circIpo11*^−/−^ mice with divergent (grey arrowheads) and convergent primers (black arrowheads). (H) *Ipo11* expression levels were detected by Western blot in *circIpo11*^*+/+*^ and *circIpo11*^−/−^ livers. β-actin was used as a loading control. *P < 0.05; **P < 0.01; *** *P* < 0.001 by two-tailed Student’s t test. Data are representative of at least three independent experiments. **Figure S4.** TOP1 interacts with *circIPO11*. (A) *CircIPO11* was predicted to interact with TOP1 via NPdock website. *CircIPO11* secondary structure was predicted according to minimum free energy (MFE). Red color indicates strong confidence of the prediction. 3D structure of TOP1 is derived from PDB database. (B) MS profiles of TOP1, corresponding peptide sequences were listed on the top of diagram. (C) *CircIPO11* and Δ*circIPO11* were overexpressed in HCC primary cells and HCC cell lines, followed by clone formation assay. Representative images (left panel), and statistical results (right panel) are shown as means ± SD. *P < 0.05 by two-tailed Student’s t test. Data are representative of at least three independent experiments. **Figure S5.** *circIPO11* overexpression recruits TOP1 on *GLI1* promoter. (A) Heat map of downstream transcription factors in *circIPO11* depleted and Ctrl HCC cells. Top 13 downregulated transcription factors were shown. (B) Different segments of *GLI1* promoter were constructed into pGL3 vector for dual luciferase reporter assays. (C) Overexpression of *circIPO11* enhanced luciferase activity. Luciferase activities were normalized to control luciferase activity. Results are shown as means ± SD. (D) ChIP-qPCR analysis of TOP1 enrichment on the − 2050 to − 1850 bp region of *GLI1* promoter in *circIPO11*-overexpressing and control cells. Indicated HCC oncosphere cells were used for ChIP assay. Results are shown as means ± SD. (E) DNase I digestion assays were performed in *circIPO11*-overexpressing and control cells. Results are shown as means ± SD. (F) TOP1 was completely deleted in TOP1 KO cells through CRISPR/Cas9 approach. β-actin was used as a loading control. (G) The binding region of TOP1 to *GLI1* promoter was confirmed by luciferase reporter assay. Results are shown as means ± SD. *P < 0.05; **P < 0.01; by two-tailed Student’s t test. Data are representative of at least three independent experiments. Representation for GLI1 knockout mice (upper panel). GLI1 deficiency was confirmed by DNA sequencing. (C) GLI1 KO efficiency in HCC cell lines were detected by Western blot. (D) GLI1-deleted or control HCC cells were transfered to 6-well plates for clone formation assay. (E) 5 × 10^5^ CSC and non-CSC cells with *GLI1*-deleted or control were subcutaneously injected into BALB/c nude mice. Results are shown as means ± SD. *n* = 4 for each group. (F) Quantitative measurement of GLI1 activation in *circIPO11*-overexpression. *n* = 3 for each group. Results are shown as means ± SD. (G) Schematic diagram of deleting the binding region of TOP1 and *GLI1* promoter by CRISPR/Cas9. Two sgRNAs were designed to target the flanking sequence of binding region. (H) *CircIPO11* overexpression had no effect on sphere formation ability by *GLI1* promoter deletion. Representative images and statistical results are shown. Scale bar, 500 μm. *P < 0.05; **P < 0.01, *** *P* < 0.001 by two-tailed Student’s t test. Data are representative of at least three independent experiments. **Figure S6.** GLI1 knockout impairs the stemness of liver CSCs. (A, B) Schematic representation for GLI1 knockout mice (upper panel). GLI1 deficiency was confirmed by DNA sequencing. (C) GLI1 KO efficiency in HCC cell lines were detected by Western blot. (D) GLI1-deleted or control HCC cells were transfered to 6-well plates for clone formation assay. (E) 5x10^5^ CSC and non-CSC cells with *GLI1*-deleted or control were subcutaneously injected into BALB/c nude mice. Results are shown as means ± SD. *n* = 4 for each group. (F) Quantitative measurement of GLI1 activation in *circIPO11*-overexpression. *n* = 3 for each group. Results are shown as means ± SD. (G) Schematic diagram of deleting the binding region of TOP1 and *GLI1* promoter by CRISPR/Cas9. Two sgRNAs were designed to target the flanking sequence of binding region. (H) *CircIPO11* overexpression had no effect on sphere formation ability by *GLI1* promoter deletion. Representative images and statistical results are shown. Scale bar, 500 μm. *P < 0.05; **P < 0.01, *** *P* < 0.001 by two-tailed Student’s t test. Data are representative of at least three independent experiments.**Figure S7.** C*ircIPO11* depletion and GLI1 knockout inhibit CSC proliferation. (A-C) *CircIPO11*, TOP1 and GLI1 mRNA levels were detected by qRT-PCR, followed by correlation analysis. Black dots represent HCC samples (*n* = 29). (D, E) CSC (CD13^+^CD133^+^) subpopulations non-CSC (CD13^−^CD133^−^) were sorted from HCC cell lines and transfected with respective vectors, followed by flow cytometry analysis. Ki-67 was stained for CSC proliferation (D), and statistical results are shown as means ± SD (E). *n* = 3 for each group. (F, G) CSC subpopulations and non-CSC were sorted from Huh7 cells and transfected with respective vectors, followed by flow cytometry analysis. 7-AAD and Annexin V was used for detection of cell death (F), and statistical results are shown as means ± SD (G). *n* = 3 for each group. **Table S1.** shRNA sequences used in this study. **Table S2.** sgRNA sequences used in this study. **Table S3.** PCR primers used in this study.

## Data Availability

All the data obtained and/or analyzed during the current study were available from the corresponding authors on reasonable request.

## Abbreviations

HCC: Hepatocellular carcinoma
CSCs: Cancer stem cells
CircRNA: Circular RNA
LncRNAs: Long noncoding RNAs
ASOs: Antisense oligonucleotides
Pre-mRNA: Precursor mRNA
DEN: Diethylnitrosamine
ORF: Open reading frame
EMSA: Electrical mobility shift assay
TFs: Transcription factors
GLI1: GLI family zinc finger 1
GSEA: Gene set enrichment analysis
TOP1: Topoisomerase 1
TSS: Transcription start site
KO: Knock out
CPT: Camptothecin
Hh: Hedgehog
PTCH1: Patched1
Shh: Sonic
Dhh: Desert
Ihh: Indian
Smo: Smoothened
RIP: RNA immunoprecipitation assay
ChIP: Chromatin immunoprecipitation assay
ChIRP: Chromatin isolation by RNA purification-PCR assay
PDC: Patient-derived tumor cells models
Poly A: Polyadenylated
RNA-seq: RNA sequencing
PLC: PLC/PRF/5
Luc: Luciferase
FISH: Fluorescent in situ hybridization
HE: Hematoxylin and Eosin
IHC: Immunohistochemistry
IF: Immunofluorescence
TOP1cc: Topoisomerase cleavage complex
