# Supplementary material for: Circular RNA circIPO11 drives self-renewal of liver cancer initiating cells via Hedgehog signaling
Source: Mol Cancer. 2021 Oct 14;20:132. doi: 10.1186/s12943-021-01435-2 (PMC8515748; doi:10.1186/s12943-021-01435-2)
Supplement: Supplementary file 1 — Additional file 1: Figure S1. Characterization of upregulated circIPO11 in HCC tumor tissues. (A) Validation of circRNAs by DNA sequencing. PCR products with divergent primers were sequenced. Divergent primers were listed in Table S3. (B) Using complementary DNA (cDNA) and genomic DNA (gDNA) as templates, circIPO11 was amplified with divergent (grey arrowheads) and convergent primers (black arrowheads). Gapdh was used as a positive control. (C) Total RNAs extracted from HCC oncospheres were digested with or without 2 U/μg RNase R for 1 h at 37 °C, followed by qPCR analysis, IPO11 and ACTB were used as positive controls. Data are shown as means ± SD. (D) HCC cell lines were treated with 2 μg/ml actinomycin D for 14 h, and whole RNAs were extracted for qPCR analysis. IPO11 and ACTB were used as positive controls. Data are shown as means ± SD. (E) Schematic representation of nine circRNAs derived from IPO11 transcript due to variable cyclizations. (F) Expression levels of the variable cycled circRNAs were examined by qRT-PCR. CircIPO11 was the only circular RNA that highly expressed in tumor, oncospheres and liver CSCs. Data are shown as means ± SD. (G) Sequence conservation analysis of circIPO11 in vertebrates from zebrafish to human. The scores listed in the table were identified by the WATER algorithm. (H, I) Conservation analysis of upstream (H) and downstream (I) complementary intron sequence of circIPO11 in vertebrates. (J) Match information of complementary introns of circIPO11 in vertebrates. U, upstream complementary intron sequences; D, downstream complementary intron sequences. **P < 0.01; *** P < 0.001 by two-tailed Student’s t test. Data are representative of at least three independent experiments. Figure S2. CircIPO11 is required for the self-renewal of liver CSCs. (A) CircIPO11 expression was detected by qRT-PCR in HCC cell lines and HCC primary tumor cells. Data are shown as means ± SD. (B, C) Expression levels of IPO11 in circIPO11-depleted HCC cells w [file 12943_2021_1435_MOESM1_ESM.docx]

**Supplementary Data**

**Supplementary Figures 1-7 and legends**

**Supplementary Tables s1-s3**


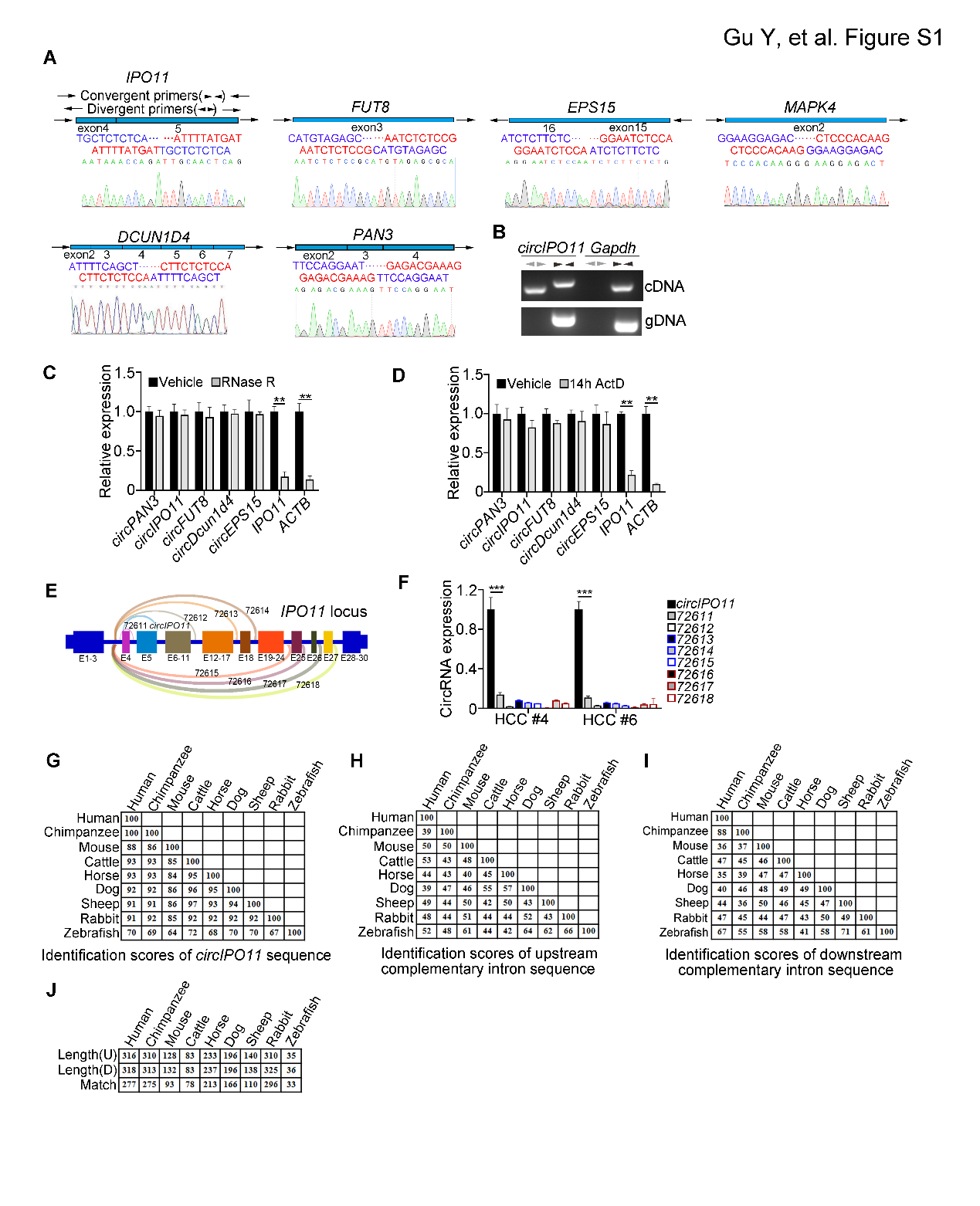


**Figure S1. Characterization of upregulated *circIPO11* in HCC tumor tissues.**

(A) Validation of circRNAs by DNA sequencing. PCR products with divergent primers were sequenced. Divergent primers were listed in Table S3. (B) Using complementary DNA (cDNA) and genomic DNA (gDNA) as templates, *circIPO11* was amplified with divergent (grey arrowheads) and convergent primers (black arrowheads). *Gapdh* was used as a positive control. (C) Total RNAs extracted from HCC oncospheres were digested with or without 2 U/μg RNase R for 1 h at 37°C, followed by qPCR analysis, *IPO11* and *ACTB* were used as positive controls. Data are shown as means ± SD. (D) HCC cell lines were treated with 2 μg/ml actinomycin D for 14 h, and whole RNAs were extracted for qPCR analysis. *IPO11* and *ACTB* were used as positive controls. Data are shown as means ± SD. (E) Schematic representation of nine circRNAs derived from *IPO11* transcript due to variable cyclizations. (F) Expression levels of the variable cycled circRNAs were examined by qRT-PCR. *CircIPO11* was the only circular RNA that highly expressed in tumor, oncospheres and liver CSCs. Data are shown as means ± SD. (G) Sequence conservation analysis of *circIPO11* in vertebrates from zebrafish to human. The scores listed in the table were identified by the WATER algorithm. (H, I) Conservation analysis of upstream (H) and downstream (I) complementary intron sequence of *circIPO11* in vertebrates. (J) Match information of complementary introns of *circIPO11* in vertebrates. U, upstream complementary intron sequences; D, downstream complementary intron sequences. ***P* < 0.01; *** *P* < 0.001 by two-tailed Student’s t test. Data are representative of at least three independent experiments.


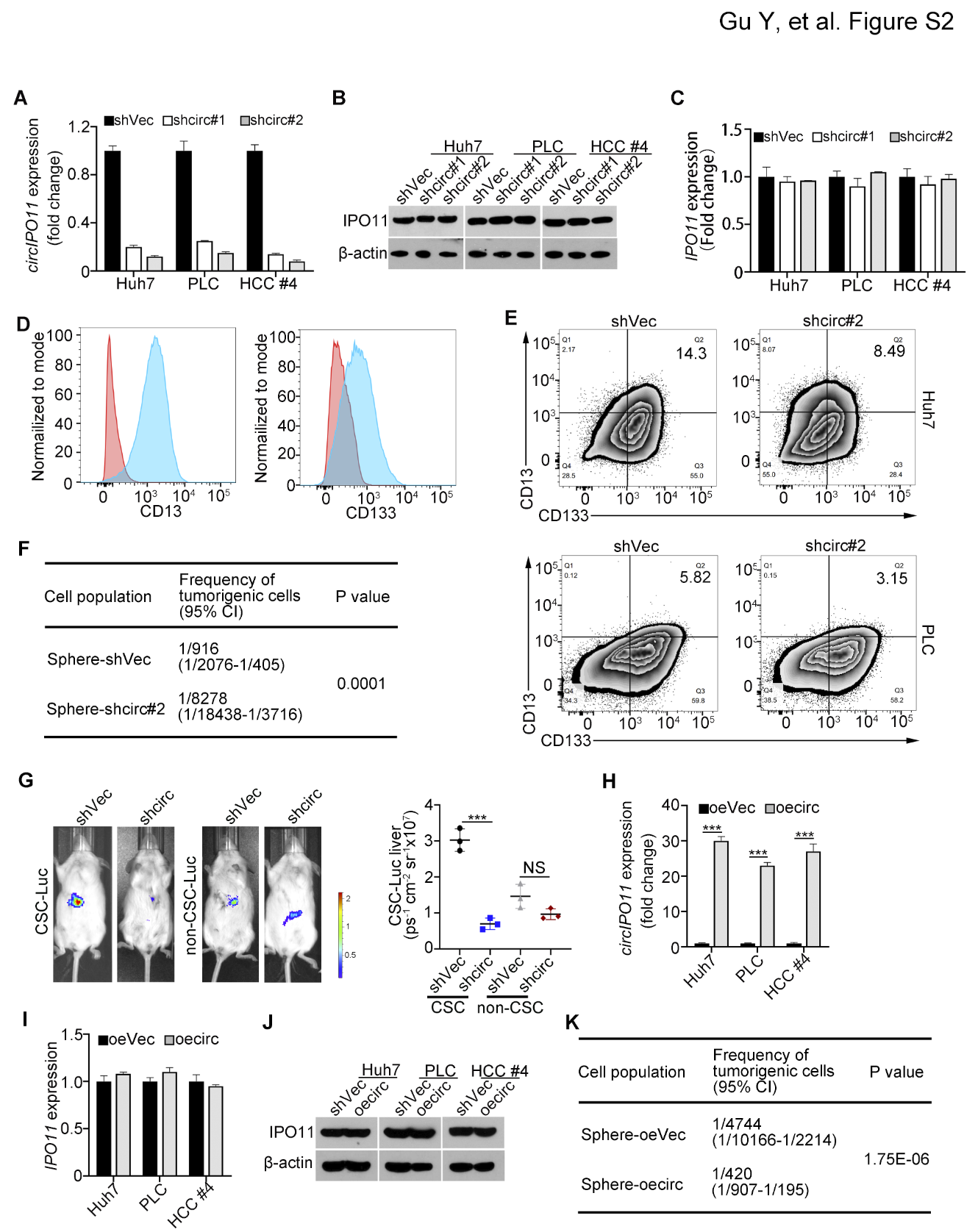


**Figure S2. *CircIPO11* is required for the self-renewal of liver CSCs.**

(A) *CircIPO11* expression was detected by qRT-PCR in HCC cell lines and HCC primary tumor cells. Data are shown as means ± SD. (B, C) Expression levels of IPO11 in *circIPO11*-depleted HCC cells were detected by Western blot (B) and qRT-PCR (C). β-actin was used as a loading control. Data are shown as means ± SD. (D) CD13 and CD133 subpopulations in HCC primary tumor cells were detected by flow cytometry. (E) CD13^+^CD133^+^ subpopulations were sorted from *circIPO11*-depleted and control HCC cell lines, followed by flow cytometry analysis. (F) Tumorigenic cell frequencies in *circIPO11*-depleted and empty vector control (shVec) cells were analyzed with a limiting dilution assay (<http://bioinf.wehi.edu.au/software/elda/>). Data are shown as means and 95% confidence intervals (n = 8). (G) Orthotopic liver tumor imaging of CSCs and non-CSCs with and without *circIPO11* transduced with Luc vectors. Representative images are shown (left panel), and statistical results are shown as means ± SD (right panel). n = 3 for each group. (H) *CircIPO11* was overexpressed in HCC cell lines and HCC primary tumor cells. Data are shown as means ± SD. (I, J) Expression of *circIPO11* did not affect the expression of its parental gene *IPO11* by qRT-PCR (I) and Western blot (J). β-actin was used as a loading control. Data are shown as means ± SD. (K) Tumorigenic cell frequencies in *circIPO11*-overexpressing and control (oeVec) cells were determined with limiting dilution assay (http://bioinf.wehi.edu.au/software/elda/). Data are shown as means and 95% confidence intervals (n = 8). *** *P* < 0.001 by two-tailed Student’s t test. Data are representative of at least three independent experiments.


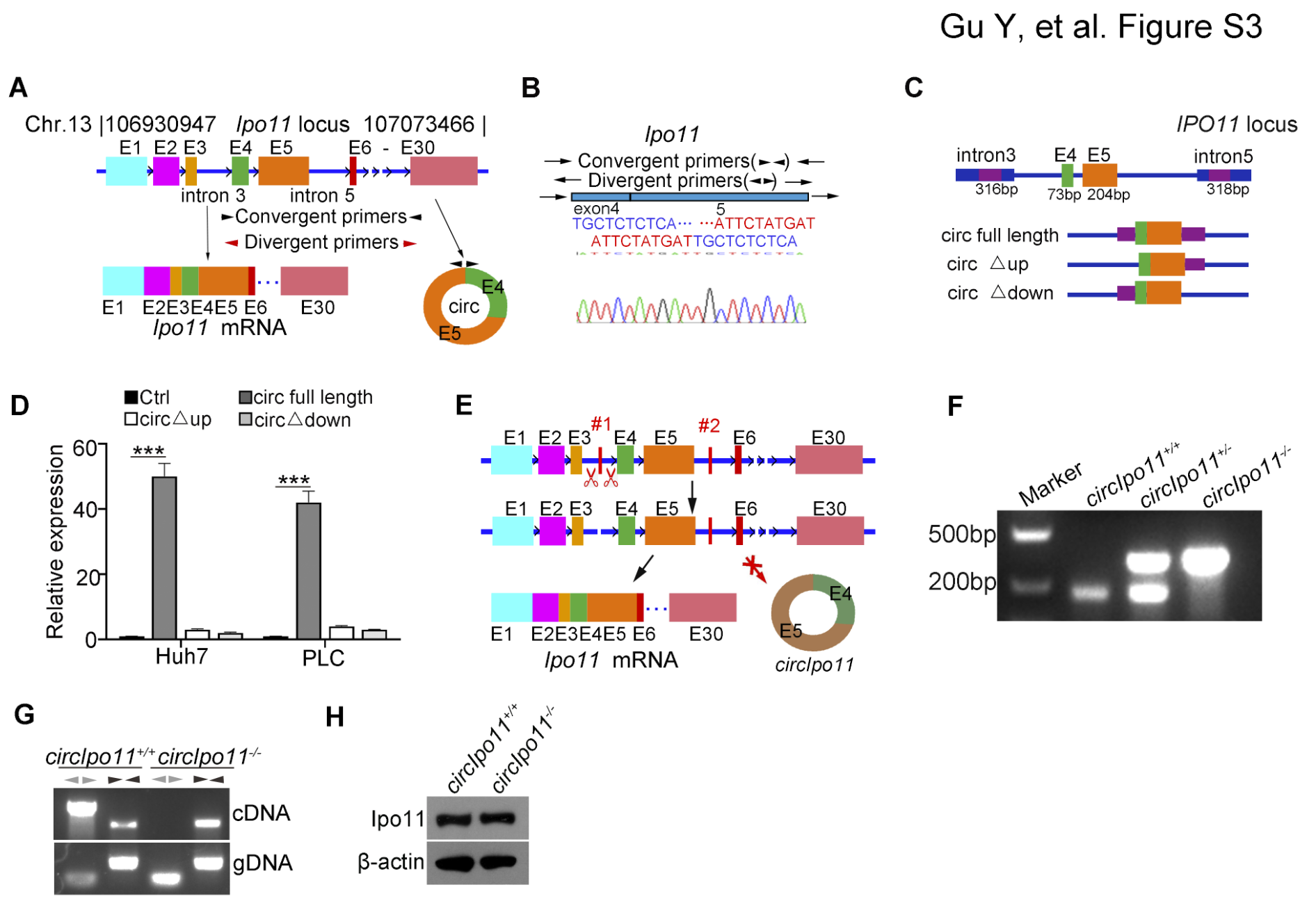


**Figure S3. Generation of *circIPO11* knockout mice.**

(A) Schematic representation of mouse *circIPO11*. Convergent primers for linear *Ipo11* were denoted in black arrowheads, and divergent primers agaisnt *circIPO11* were denoted in red arrowheads. E1, exon 1. (B) Validation of *circIPO11* by DNA sequencing in mice. PCR products with divergent primers were sequenced. (C, D) Schematic representation of mini-gene assay was shown (C). circ△up: missing upstream intronic complementary sequences flanking exon 4. circ△down: missing downstream intronic complementary sequences flanking exon 5. Identification of complementary intron sequences were required for *circIPO11* formation, followed by qRT-PCR (D). (E) Schematic representation for *circIpo11* KO mice. The upstream complementary sequences (#1) were deleted using CRISPR/Cas9 system. Corresponding sgRNAs were shown in Table S2. (F) *CircIpo11^-/-^* mice were verified by agarose gel electrophoresis. Genome DNA of *circIpo11^+/+^* and *circIpo11**^-/-^* mice was used for PCR template. WT allele had a PCR length of about 222 bp and deficient allele had a PCR length of about 336 bp. (G) Using complementary DNA (cDNA) and genomic DNA (gDNA) as templates, *circIpo11* was detected in WT or *circIpo11^-/-^* mice with divergent (grey arrowheads) and convergent primers (black arrowheads). (H) *Ipo11* expression levels were detected by Western blot in *circIpo11^+/+^* and *circIpo11^-/-^* livers. β-actin was used as a loading control. **P* < 0.05; ***P* < 0.01; *** *P* < 0.001 by two-tailed Student’s t test. Data are representative of at least three independent experiments.


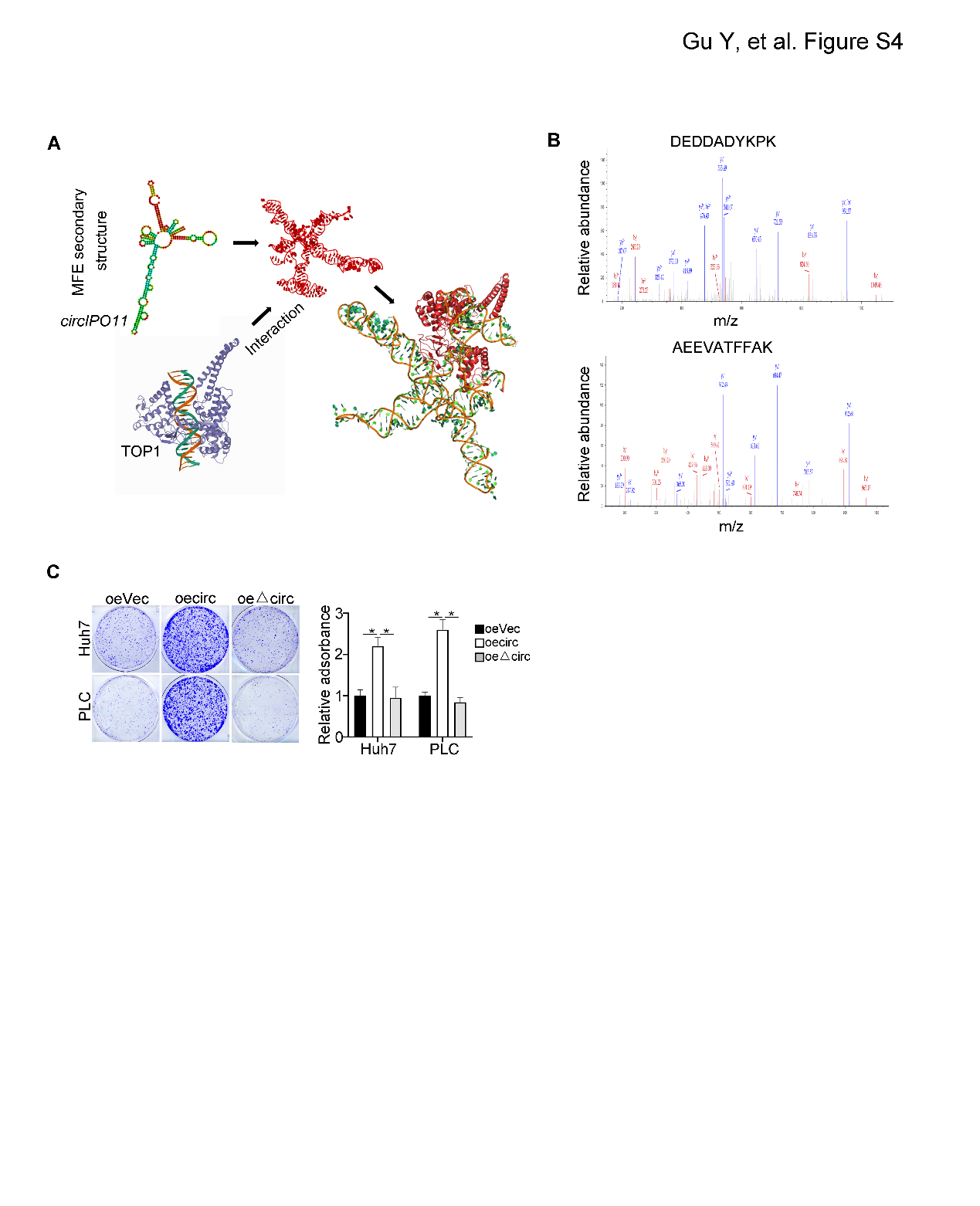


**Figure S4. TOP1 interacts with *circIPO11*.**

(A) *CircIPO11* was predicted to interact with TOP1 via NPdock website. *CircIPO11* secondary structure was predicted according to minimum free energy (MFE). Red color indicates strong confidence of the prediction. 3D structure of TOP1 is derived from PDB database. (B) MS profiles of TOP1, corresponding peptide sequences were listed on the top of diagram. (C) *CircIPO11* and Δ*circIPO11* were overexpressed in HCC primary cells and HCC cell lines, followed by clone formation assay. Representative images (left panel), and statistical results (right panel) are shown as means ± SD. **P* < 0.05 by two-tailed Student’s t test. Data are representative of at least three independent experiments.


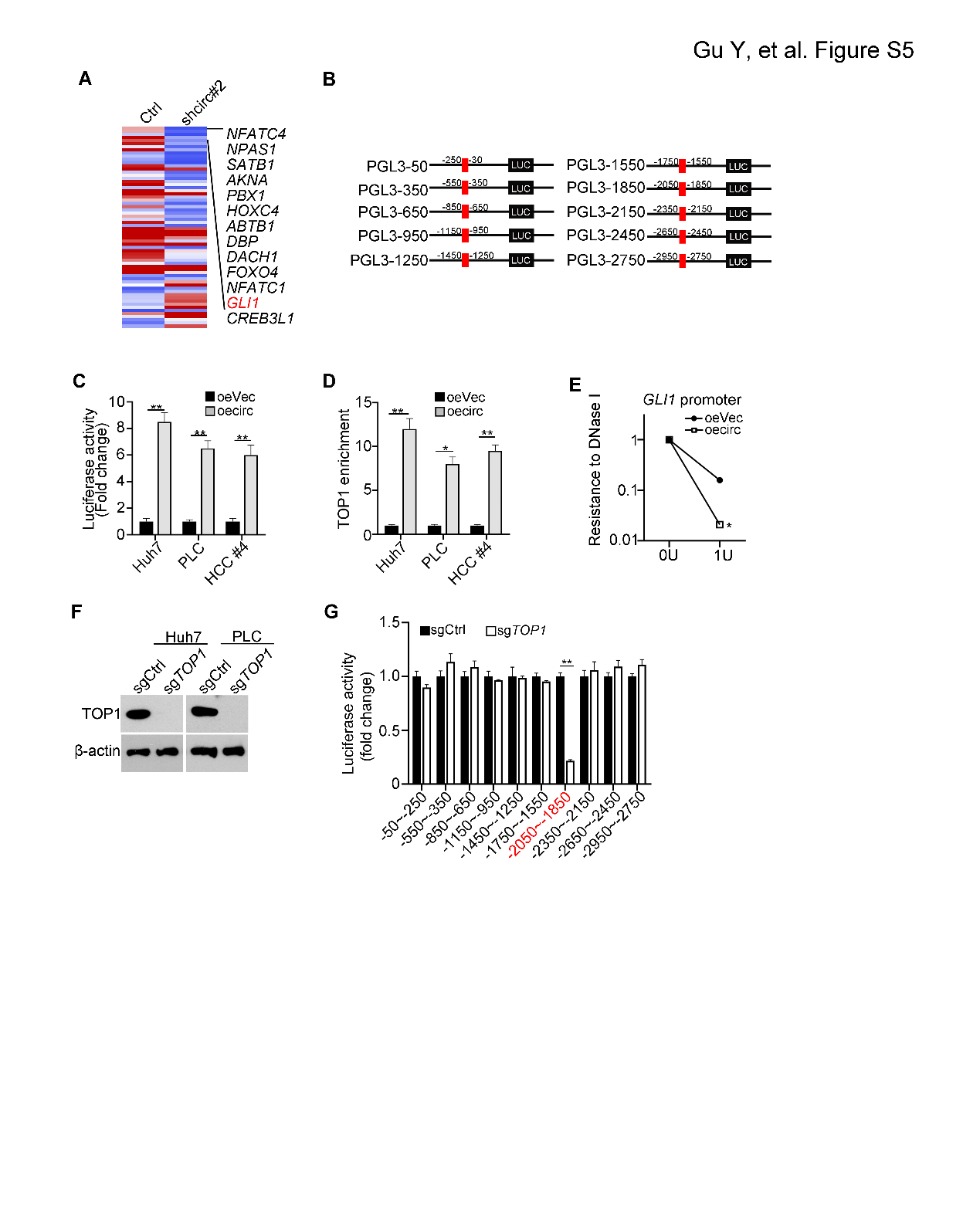


**Figure S5. *circIPO11* overexpression recruits TOP1 on *GLI1* promoter.**

(A) Heat map of downstream transcription factors in *circIPO11* depleted and Ctrl HCC cells. Top 13 downregulated transcription factors were shown. (B) Different segments of *GLI1* promoter were constructed into pGL3 vector for dual luciferase reporter assays. (C) Overexpression of *circIPO11* enhanced luciferase activity. Luciferase activities were normalized to control luciferase activity. Results are shown as means ± SD. (D) ChIP-qPCR analysis of TOP1 enrichment on the -2050 to -1850 bp region of *GLI1* promoter in *circIPO11*-overexpressing and control cells. Indicated HCC oncosphere cells were used for ChIP assay. Results are shown as means ± SD. (E) DNase I digestion assays were performed in *circIPO11*-overexpressing and control cells. Results are shown as means ± SD. (F) TOP1 was completely deleted in TOP1 KO cells through CRISPR/Cas9 approach. β-actin was used as a loading control. (G) The binding region of TOP1 to *GLI1* promoter was confirmed by luciferase reporter assay. Results are shown as means ± SD. **P* < 0.05; ***P* < 0.01; by two-tailed Student’s t test. Data are representative of at least three independent experiments.


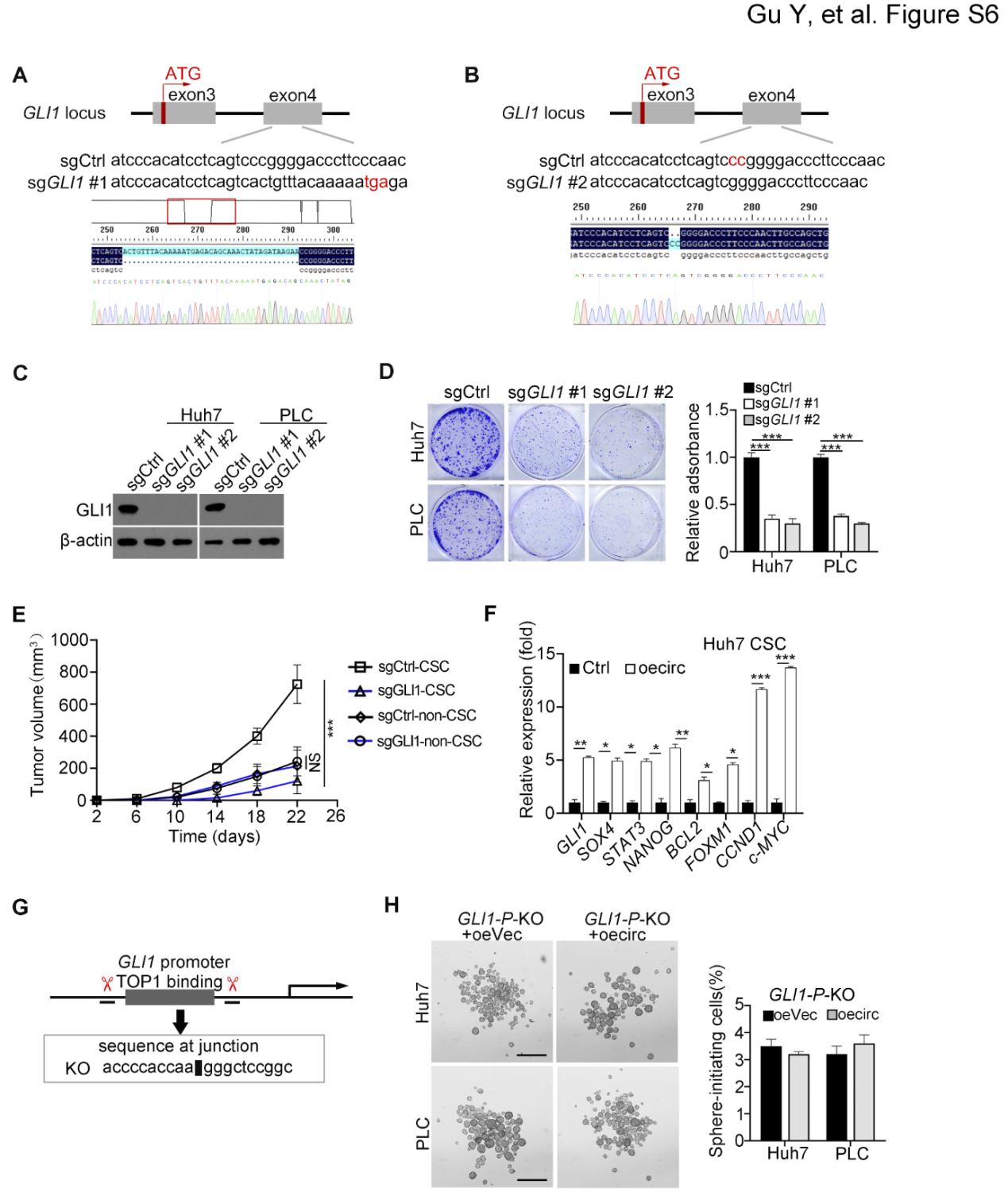


**Figure S6. GLI1 knockout impairs the stemness of liver CSCs.**

(A, B) Schematic representation for GLI1 knockout mice (upper panel). GLI1 deficiency was confirmed by DNA sequencing. (C) GLI1 KO efficiency in HCC cell lines were detected by Western blot. (D) GLI1-deleted or control HCC cells were transfered to 6-well plates for clone formation assay. (E) 5x10^5^ CSC and non-CSC cells with *GLI1*-deleted or control were subcutaneously injected into BALB/c nude mice. Results are shown as means ± SD. n = 4 for each group. (F) Quantitative measurement of GLI1 activation in *circIPO11*-overexpression. *n* = 3 for each group. Results are shown as means ± SD. (G) Schematic diagram of deleting the binding region of TOP1 and *GLI1* promoter by CRISPR/Cas9. Two sgRNAs were designed to target the flanking sequence of binding region. (H) *CircIPO11* overexpression had no effect on sphere formation ability by *GLI1* promoter deletion. Representative images and statistical results are shown. Scale bar, 500 μm. **P* < 0.05; ***P* < 0.01, *** *P* < 0.001 by two-tailed Student’s t test. Data are representative of at least three independent experiments.


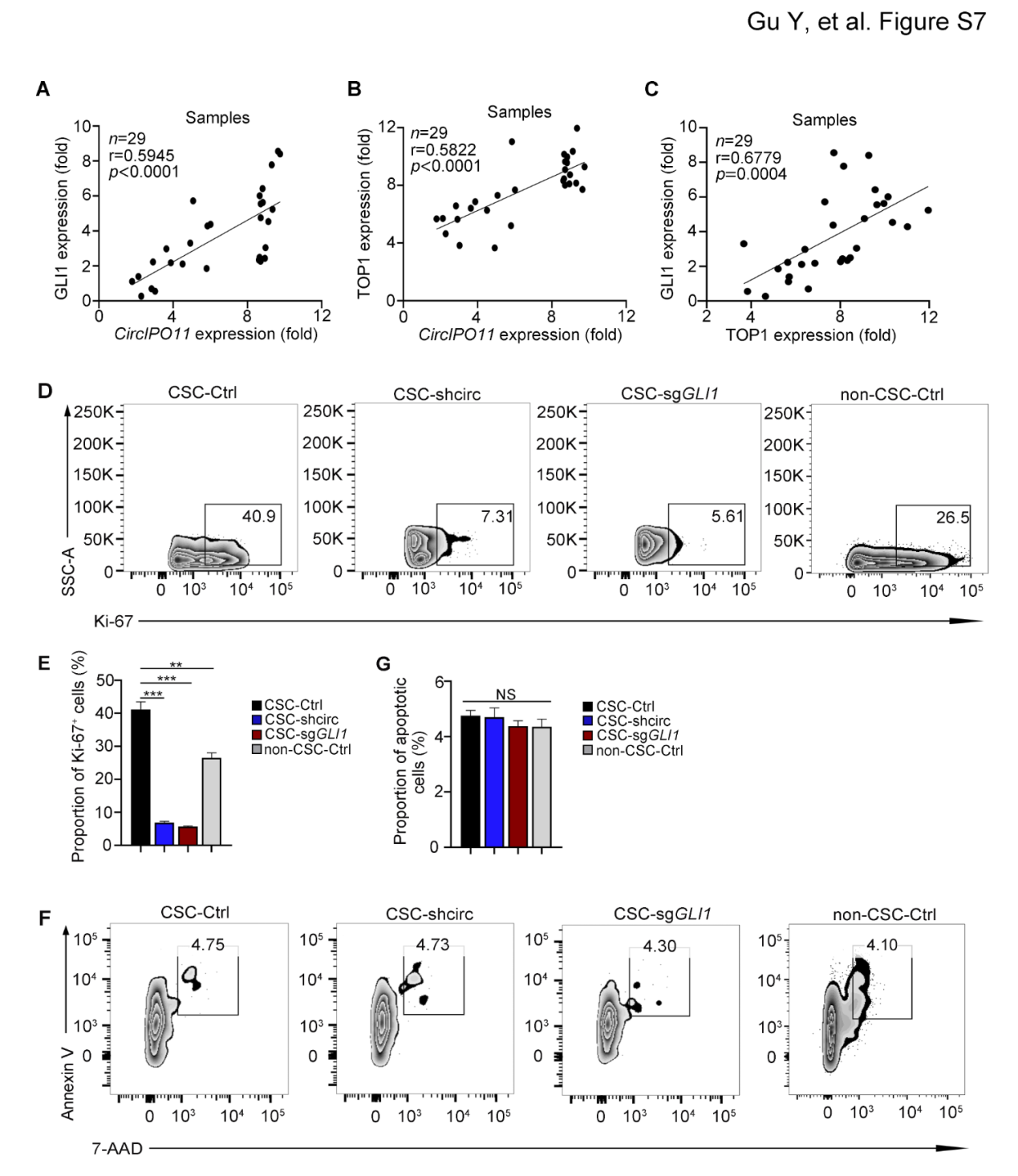


**Figure S7. C*ircIPO11* depletion and GLI1 knockout inhibit CSC proliferation.** (A-C) *CircIPO11*, TOP1 and GLI1 mRNA levels were detected by qRT-PCR, followed by correlation analysis. Black dots represent HCC samples (n=29). (D, E) CSC (CD13^+^CD133^+^) subpopulations non-CSC (CD13^-^CD133^-^) were sorted from HCC cell lines and transfected with respective vectors, followed by flow cytometry analysis. Ki-67 was stained for CSC proliferation (D), and statistical results are shown as means ± SD (E). n = 3 for each group. (F, G) CSC subpopulations and non-CSC were sorted from Huh7 cells and transfected with respective vectors, followed by flow cytometry analysis. 7-AAD and Annexin V was used for detection of cell death (F), and statistical results are shown as means ± SD (E). n = 3 for each group.

**Table S1. shRNA sequences used in this study**

| shRNA | Sequences |
| --- | --- |
| *CircPAN3-1#* | 5’-TTCCAGGAATGGATGGAGG-3’ |
| *CircPAN3-2#* | 5’-AGGAATGGATGGAGGTGCT-3' |
| *CircIPO11-1#* | 5’-AAACTATTTTATGATTGCT-3' |
| *CircIPO11-2#* | 5’-TGATTGCTCTCTCAGAGGA-3' |
| *CircFUT8-1#* | 5’-gccgaatctctccgcatgt-3’ |
| *CircFUT8-2#* | 5’-ctccgcatgtagagcgcat-3' |
| *CircDcun1d4-1#* | 5’-[CTCCAATTTTCAGCTGAAC](http://bioinfo.clontech.com/rnaidesigner/oligoDesigner.do?senseSequence=CTCCAATTTTCAGCTGAAC&restrictionSite=yes&overhangs=yes&loopSequence=TTCAAGAGA)-3’ |
| *CircDcun1d4-2#* | 5’-[GAATGACTTCTCTCCAATT](http://bioinfo.clontech.com/rnaidesigner/oligoDesigner.do?senseSequence=GAATGACTTCTCTCCAATT&restrictionSite=yes&overhangs=yes&loopSequence=TTCAAGAGA)-3' |
| *CircEPS15-1#* | 5’-[CCAGGAATCTCCAATCTCT](http://bioinfo.clontech.com/rnaidesigner/oligoDesigner.do?senseSequence=CCAGGAATCTCCAATCTCT&restrictionSite=yes&overhangs=yes&loopSequence=TTCAAGAGA)-3’ |
| *CircEPS15-2#* | 5’-[CAGGAATCTCCAATCTCTT](http://bioinfo.clontech.com/rnaidesigner/oligoDesigner.do?senseSequence=CAGGAATCTCCAATCTCTT&restrictionSite=yes&overhangs=yes&loopSequence=TTCAAGAGA)-3' |
| *CircMAPK4-1#* | 5’-CAAGGGAAGGAGACTTGGT-3’ |
| *CircMAPK4-2#* | 5’-GGAAGGAGACTTGGTCTGA-3’ |
| *NFATC4-1#* | 5’-[TCACGAATCTCCCATCTAA](http://bioinfo.clontech.com/rnaidesigner/oligoDesigner.do?senseSequence=TCACGAATCTCCCATCTAA&restrictionSite=yes&overhangs=yes&loopSequence=TTCAAGAGA)-3’ |
| *NFATC4-2#* | 5’-[GGATGAGGAGCTGGAATTT](http://bioinfo.clontech.com/rnaidesigner/oligoDesigner.do?senseSequence=GGATGAGGAGCTGGAATTT&restrictionSite=yes&overhangs=yes&loopSequence=TTCAAGAGA)-3’ |
| *NPAS1-1#* | 5’-[TGGACACATGATCGTCTTC](http://bioinfo.clontech.com/rnaidesigner/oligoDesigner.do?senseSequence=TGGACACATGATCGTCTTC&restrictionSite=yes&overhangs=yes&loopSequence=TTCAAGAGA)-3' |
| *NPAS1-2#* | 5’-[GTCTCACCATCCTTGCTTG](http://bioinfo.clontech.com/rnaidesigner/oligoDesigner.do?senseSequence=GTCTCACCATCCTTGCTTG&restrictionSite=yes&overhangs=yes&loopSequence=TTCAAGAGA)-3' |
| *SATB1-1#* | 5’-[CAGATATGCTTCAAGATGT](http://bioinfo.clontech.com/rnaidesigner/oligoDesigner.do?senseSequence=CAGATATGCTTCAAGATGT&restrictionSite=yes&overhangs=yes&loopSequence=TTCAAGAGA)-3' |
| *SATB1-2#* | 5’-[GTCACATTGAAAATTCAGT](http://bioinfo.clontech.com/rnaidesigner/oligoDesigner.do?senseSequence=GTCACATTGAAAATTCAGT&restrictionSite=yes&overhangs=yes&loopSequence=TTCAAGAGA)-3’ |
| *AKNA-1#* | 5’-[GGACAAACTTTCTGAACAT](http://bioinfo.clontech.com/rnaidesigner/oligoDesigner.do?senseSequence=GGACAAACTTTCTGAACAT&restrictionSite=yes&overhangs=yes&loopSequence=TTCAAGAGA)-3' |
| *AKNA-2#* | 5’-[AGACAAAGACGTCACCTAA](http://bioinfo.clontech.com/rnaidesigner/oligoDesigner.do?senseSequence=AGACAAAGACGTCACCTAA&restrictionSite=yes&overhangs=yes&loopSequence=TTCAAGAGA)-3’ |
| *PBX1-1#* | 5’-[GGAGCATTCAGATTACAGA](http://bioinfo.clontech.com/rnaidesigner/oligoDesigner.do?senseSequence=GGAGCATTCAGATTACAGA&restrictionSite=yes&overhangs=yes&loopSequence=TTCAAGAGA)-3' |
| *PBX1-2#* | 5’-[GCTGGACAACATGCTGTTA](http://bioinfo.clontech.com/rnaidesigner/oligoDesigner.do?senseSequence=GCTGGACAACATGCTGTTA&restrictionSite=yes&overhangs=yes&loopSequence=TTCAAGAGA)-3’ |
| *HOXC4-1#* | 5’-[GCAGCAAGTCCTGGAATTA](http://bioinfo.clontech.com/rnaidesigner/oligoDesigner.do?senseSequence=GCAGCAAGTCCTGGAATTA&restrictionSite=yes&overhangs=yes&loopSequence=TTCAAGAGA)-3' |
| *HOXC4-2#* | 5’-[TCCAAAACCGTCGCATGAA](http://bioinfo.clontech.com/rnaidesigner/oligoDesigner.do?senseSequence=TCCAAAACCGTCGCATGAA&restrictionSite=yes&overhangs=yes&loopSequence=TTCAAGAGA)-3’ |
| *ABTB1-1#* | 5’-[GTGTGAAGGTGCTGACCAT](http://bioinfo.clontech.com/rnaidesigner/oligoDesigner.do?senseSequence=GTGTGAAGGTGCTGACCAT&restrictionSite=yes&overhangs=yes&loopSequence=TTCAAGAGA)-3’ |
| *ABTB1-1#* | 5’-[CTGACATCTGCTTCCGAG](http://bioinfo.clontech.com/rnaidesigner/oligoDesigner.do?senseSequence=CTGACATCTGCTTCCGAGT&restrictionSite=yes&overhangs=yes&loopSequence=TTCAAGAGA)T-3’ |
| *DBP-1#* | 5’-[AGCTGATCTTGCCCTATCA](http://bioinfo.clontech.com/rnaidesigner/oligoDesigner.do?senseSequence=AGCTGATCTTGCCCTATCA&restrictionSite=yes&overhangs=yes&loopSequence=TTCAAGAGA)-3’ |
| *DBP-2#* | 5’-[TTCTCAGAAGAGGAACTTA](http://bioinfo.clontech.com/rnaidesigner/oligoDesigner.do?senseSequence=TTCTCAGAAGAGGAACTTA&restrictionSite=yes&overhangs=yes&loopSequence=TTCAAGAGA) -3' |
| *DACH1-1#* | 5’-[TCAGAATAATGAGTGCAA](http://bioinfo.clontech.com/rnaidesigner/oligoDesigner.do?senseSequence=TCAGAATAATGAGTGCAAA&restrictionSite=yes&overhangs=yes&loopSequence=TTCAAGAGA)A-3' |
| *DACH1-2#* | 5’-[TGCATACGGTCTACACCAA](http://bioinfo.clontech.com/rnaidesigner/oligoDesigner.do?senseSequence=TGCATACGGTCTACACCAA&restrictionSite=yes&overhangs=yes&loopSequence=TTCAAGAGA)-3' |
| *FOXO4-1#* | 5’-[GCAAAAGCTCTTGGTGGAT](http://bioinfo.clontech.com/rnaidesigner/oligoDesigner.do?senseSequence=GCAAAAGCTCTTGGTGGAT&restrictionSite=yes&overhangs=yes&loopSequence=TTCAAGAGA)-3’ |
| *FOXO4-2#* | 5’-[CGTGAAGAAGCCGATATGT](http://bioinfo.clontech.com/rnaidesigner/oligoDesigner.do?senseSequence=CGTGAAGAAGCCGATATGT&restrictionSite=yes&overhangs=yes&loopSequence=TTCAAGAGA)-3' |
| *NFATC1-1#* | 5’-[[CCTGTACCACAACAATAAC](http://bioinfo.clontech.com/rnaidesigner/oligoDesigner.do?senseSequence=CCTGTACCACAACAATAAC&restrictionSite=yes&overhangs=yes&loopSequence=TTCAAGAGA)](http://bioinfo.clontech.com/rnaidesigner/oligoDesigner.do?senseSequence=CTCCAATTTTCAGCTGAAC&restrictionSite=yes&overhangs=yes&loopSequence=TTCAAGAGA)-3’ |
| *NFATC1-2#* | 5’-[TGGCTATGCATCCTCCAAC](http://bioinfo.clontech.com/rnaidesigner/oligoDesigner.do?senseSequence=TGGCTATGCATCCTCCAAC&restrictionSite=yes&overhangs=yes&loopSequence=TTCAAGAGA)-3' |
| *GLI1-1#* | 5’-GCCGTATGTATGTAAGCTC-3’ |
| *GLI1-2#* | 5’-[GTCATACTCACGCCTCGAA](http://bioinfo.clontech.com/rnaidesigner/oligoDesigner.do?senseSequence=GTCATACTCACGCCTCGAA&restrictionSite=yes&overhangs=yes&loopSequence=TTCAAGAGA)-3' |
| *CREB3L1-1#* | 5’-[GTCGGACTTCCTCAACAAT](http://bioinfo.clontech.com/rnaidesigner/oligoDesigner.do?senseSequence=GTCGGACTTCCTCAACAAT&restrictionSite=yes&overhangs=yes&loopSequence=TTCAAGAGA)-3’ |
| *CREB3L1-2#* | 5’-[CGGAGAACATGGAGGACTT](http://bioinfo.clontech.com/rnaidesigner/oligoDesigner.do?senseSequence=CGGAGAACATGGAGGACTT&restrictionSite=yes&overhangs=yes&loopSequence=TTCAAGAGA)-3’ |
| ASO1 | mU*mC*mC*mT*mC*T*G*A*G*A*G*A*G*C*A*mA*mU*mC*mA*mU |
| ASO2 | mU*mG*mA*mG*mA*G*A*G*C*A*A*T*C*A*T*mA*mA*mA*mA*mU |

**Table S2. sgRNA sequences used in this study**

| sgRNA | Sequences |
| --- | --- |
| *circIpo11* (knockout)  *GLI1*  *TOP1*  *GLI1p* | 5’-CTATAGTACAGGGCAACAGCAGG-3’  5’- GTGGCACCTGTGAGAGGCGGAGG-3’  5’-CATGACTCTGCCCGGGGTG-3’  5’-ATCCCACATCCTCAGTCCCG-3’  5’-ACACAAAGATCGAGAACAC-3’  5’-CGGCACAAAGAACACAAGA-3’  5’-CACCAACGGGGACCCCCACTC-3’  5’-AGATGGTTTAGGGCTCCGGC-3’ |

*GLI1p*: *GLI1* promoter

**Table S3. PCR primers used in this study**

| Primers | Sequences |
| --- | --- |
| *18S (Forward)* | 5’-AACCCGTTGAACCCCATT-3’ |
| *18S (Reverse)* | 5’-CCATCCAATCGGTAGTAGCG-3’ |
| *CircIPO11 (Convergent Forward)* | 5’-GAGGAGAAAACTACTCTGCGTG-3’ |
| *CircIPO11 (Convergent Reverse)* | 5’-ATTAGTTCAGGCCACTGTCTGG-3’ |
| *CircIPO11 (Divergent Forward)* | 5’-TCGACAGCACAGAGCATTAC-3’ |
| *CircIPO11 (Divergent Reverse)* | 5’-ATTGAAGTTGGTGATGAGCCCT-3’ |
| *ACTB (Forward)* | 5’-GTCACCAACTGGGACGACAT-3’ |
| *ACTB (Reverse)* | 5’-AGGGATAGCACAGCCTGGAT-3’ |
| *GAPDH (Forward)* | 5’-ATCAGCAATGCCTCCTGCAC-3’ |
| *GAPDH (Reverse)* | 5’-TGGCATGGACTGTGGTCATG-3’ |
| *HMBS (Forward)* | 5’-AGCTTGCTCGCATACAGACG-3’ |
| *HMBS (Reverse)* | 5’-AGCTCCTTGGTAAACAGGCTT-3’ |
| *IPO11 (Forward)* | 5’-TGCAGGGCTCATCACCAAC-3’ |
| *IPO11 (Reverse)* | 5’-GCAAGTCGTTTAGATGCCAGTG-3’ |
| *Circipo11 KO (Forward)* | 5’- gcttgacttcaccatggtcc-3’ |
| *Circipo11 KO (Reverse1)* | 5’- tctggcttctgtgacaccat-3’ |
| *Circipo11 KO (Reverse2)* | 5’- gccaatcagctttctcctaga-3’ |
| *Circipo11 (Convergent Forward)* | 5’-GCAGGGCTCATCACCAACT-3’ |
| *Circipo11 (Convergent Reverse)* | 5’-CGAGCAACCTTGGCAATCAG-3’ |
| *Circipo11 (Divergent Forward)* | 5’-TCTACCATGTCACCAAGACGC-3’ |
| *Circipo11 (Divergent Reverse)* | 5’-CTAACCGAGCAACCTTGGCA-3’ |
| *NFATC4 (Forward)* | 5’-CTTCTCCGATGCCTCTGACG-3’ |
| *NFATC4 (Reverse)* | 5’- CGGGGCTTGGACCATACAG-3’ |
| *NPAS1 (Forward)* | 5’-TCTTTGTCCGCATGAAATCCA-3’ |
| *NPAS1 (Reverse)* | 5’-GCTGAGACGGAAGACGATCA-3’ |
| *SATB1 (Forward)* | 5’-GATCATTTGAACGAGGCAACTCA-3’ |
| *SATB1 (Reverse)* | 5’-TGGACCCTTCGGATCACTCA-3’ |
| *AKNA (Forward)* | 5’-CCCAGCAAGTTCCCATGAG-3’ |
| *AKNA (Reverse)* | 5’-TCATACCCCAACCTTGAGGAG-3’ |
| *PBX1 (Forward)* | 5’-GACAACTCAGTGGAGCATTCA-3’ |
| *PBX1 (Reverse)* | 5’-CTCTCGCAGGAGATTCATCAC-3’ |
| *HOXC4 (Forward)* | 5’- GAGCGCCAGTATAGCTGCAC-3’ |
| *HOXC4 (Reverse)* | 5’- GCGACTGTGATTTCTCGGGG-3’ |
| *ABTB1 (Forward)* | 5’-GTTTGTGGCGTCTAAGCCAG-3’ |
| *ABTB1 (Reverse)* | 5’-CAGCTCCCAAAGATCACCTCG-3’ |
| *DBP (Forward)* | 5’-CTGATCTTGCCCTATCAAGCATT-3’ |
| *DBP (Reverse)* | 5’-CGATGTCTTCGAGGGTCAAAG-3’ |
| *DACH1 (Forward)* | 5’- ATGTGGAACAAGTTCGCATCC-3’ |
| *DACH1 (Reverse)* | 5’- TGCAGTCATTGTAGAGGGTCT-3’ |
| *FOXO4 (Forward)* | 5’- GGCTGCCGCGATCATAGAC-3’ |
| *FOXO4 (Reverse)* | 5’- GGCTGGTTAGCGATCTCTGG-3’ |
| *NFATC1 (Forward)* | 5’- TGTGCCGGAATCCTGAAACTC-3’ |
| *NFATC1 (Reverse)* | 5’- GAGCATTCGATGGGGTTGGAG-3’ |
| *GLI1 (Forward)* | 5’-ATGTTCAACTCGATGACCCCA-3’ |
| *GLI1 (Reverse)* | 5’-GGCACTAGAGTTGAGGAATTCTGT-3’ |
| *CREB3L1 (Forward)* | 5’- GCACCTGGACCACTTTACGG-3’ |
| *CREB3L1 (Reverse)* | 5’- AGCACAGGGTCATCAAAGAAG-3’ |
| *CircDcun1d4 (Divergent Forward)* | 5’-CCTAGCTTGGAAATTGGATGCAC-3’ |
| *CircDcun1d4 (Divergent Reverse)* | 5’-CCGCAGACTTCCTGTTTGGT-3’ |
| *CircZFR (Divergent Forward)* | 5’-CTCGTGCTCAAGATTGGGGAT-3’ |
| *CircZFR (Divergent Reverse)* | 5’-TCTCCTTCCCTTGTGGGAGT-3’ |
| *CircFUT8 (Divergent Forward)* | 5’-TGGTTCCTGGCGTTGGATTA-3’ |
| *CircFUT8 (Divergent Reverse)* | 5’-GAGATCCTCCTGGTGATATGTGT-3’ |
| *CircPAN3 (Divergent Forward)* | 5’-TGCCAAGCCATATTCAGCCC-3’ |
| *CircPAN3 (Divergent Reverse)* | 5’-AAAGCACCTCCATCCATTCCTG-3’ |
| *CircEPS15 (Divergent Forward)* | 5’-AGCAGTGAAACAGCCAACCTTA-3’ |
| *CircEPS15 (Divergent Reverse)* | 5’-CACTCTCCTCCAATTCTGCTGT-3’ |
| *Circ72611 (Forward)* | 5’-ACCAACTTCAATGAACCAAT-3’ |
| *Circ72611 (Reverse)* | 5’-CAGAGTAGTTTTCTCCTCC-3’ |
| *Circ72612 (Forward)* | 5’-ATAGCAGCCCTGAAACTC-3’ |
| *Circ72612 (Reverse)* | 5’-CACTGTCTGGGACAATCC-3’ |
| *Circ72613 (Forward)* | 5’-CAAGATTTAGTGGTCCGTAT-3’ |
| *Circ72613 (Reverse)* | 5’-AGTGGGAATTAGTTCAGG-3’ |
| *Circ72614 (Forward)* | 5’-GGCTCATCACCAACTTCA-3’ |
| *Circ72614 (Reverse)* | 5’-CACTGTCTGGGACAATCC-3’ |
| *Circ72615 (Forward)* | 5’-CAGTAGGTCTATGCCAGTC-3’ |
| *Circ72615 (Reverse)* | 5’-TGAAGTTGGTGATGAGCC-3’ |
| *Circ72616 (Forward)* | 5’-GGCTCATCACCAACTTCA-3’ |
| *Circ72616 (Reverse)* | 5’-CACTGTCTGGGACAATCC-3’ |
| *Circ72617 (Forward)* | 5’-ATCTTGGAGTTATGGGTC-3’ |
| *Circ72617 (Reverse)* | 5’-TGAAGTTGGTGATGAGCC-3’ |
| *Circ72618 (Forward)* | 5’-GATCGAATGGACAACATTAC-3’ |
| *Circ72618 (Reverse)* | 5’-GTTGATCGAATGGACAACAT-3’ |
| *-250 (Forward) (GLI1p)* | 5’-TGACCTCAGGTGATCCTCCCG-3’ |
| *-50 (Reverse) (GLI1p)* | 5’-AAAGGTGGAGGAAATAGAGACTGTCAGA-3’ |
| *-550 (Forward) (GLI1p)* | 5’- AGAGTACACAGACTACAAGACTGCCT -3’ |
| *-350 (Reverse) (GLI1p)* | 5’- GAAGGCTGAGGCAGAAGAATCGC -3’ |
| *-850 (Forward) (GLI1p)* | 5’- TGCTGGACACTGCAGAGATTGTAC -3’ |
| *-650 (Reverse) (GLI1p)* | 5’- TTAACTTCAAGTGATTTTAGTAACATGGAC -3’ |
| *-1150 (Forward) (GLI1p)* | 5’- GCACCTTCTTCCTCGCTACGC -3’ |
| *-950 (Reverse) (GLI1p)* | 5’- TAAGGTTCCTGAGGGGAGTCTTCTG -3’ |
| *-1450 (Forward) (GLI1p)* | 5’-GATCCGCACCTCGGTTGGAAAA-3’ |
| *-1250 (Reverse) (GLI1p)* | 5’-GGGATGCTGGGATTTAGGGTGAG-3’ |
| *-1750 (Forward) (GLI1p)* | 5’- GCTAAAAAAGTTGGCGACATTTTCC -3’ |
| *-1550 (Reverse) (GLI1p)* | 5’- GGGAAGTTTGCAAATGATCCCCC -3’ |
| *-2050 (Forward) (GLI1p)* | 5’- CGCCCCTCAGCCCCCA -3’ |
| *-1850 (Reverse) (GLI1p)* | 5’- CAGCGTCCCCCTCGCA -3’ |
| *-2350 (Forward) (GLI1p)* | 5’- TATCTCCCTGGCATCCCCGC -3’ |
| *-2150 (Reverse) (GLI1p)* | 5’- AAAGGGAGAAGAATCCAGAAAGTATAGGG -3’ |
| *-2650 (Forward) (GLI1p)* | 5’- CTCTGGCTCAGACCACCCTG-3’ |
| *-2450 (Reverse) (GLI1p)* | 5’- TACTGGAACGTAAAGACCTATCCTAG -3’ |
| *-2950 (Forward) (GLI1p)* | 5’- AAAGGAGTGTCTTCTGGACTTGGAA -3’ |
| *-2750 (Reverse) (GLI1p)* | 5’- CTGCGGCTGGCTCCCG-3’ |
| *Nanog (Forward)* | 5’-TTTGTGGGCCTGAAGAAAACT-3’ |
| *Nanog (Reverse)* | 5’-AGGGCTGTCCTGAA TAAGCAG-3’ |
| *SMO (Forward)* | 5’-GAAGTGCCCTTGGTTCGGA-3’ |
| *SMO (Reverse)* | 5’-GCAGGGTAGCGATTCGAGTT-3’ |
| *PTCH1 (Forward)* | 5’-CCACAGAAGCGCTCCTACA-3’ |
| *PTCH1 (Reverse)* | 5’-CTGTAATTTCGCCCCTTCC-3’ |
| *TOP1 (Forward)* | 5’-ATGAGTGGGGACCACCTCCA-3’ |
| *TOP1 (Reverse)* | 5’-TCATAGTCTTCATCAGCCATGTCAAT-3’ |
| *SOX4 (Forward)* | 5’-AGCGACAAGATCCCTTTCATTC-3’ |
| *SOX4 (Reverse)* | 5’-CGTTGCCGGACTTCACCTT-3’ |
| *STAT3 (Forward)* | 5’-CAGCAGCTTGACACACGGTA-3’ |
| *STAT3 (Reverse)* | 5’-AAACACCAAAGTGGCATGTGA-3’ |
| *BCL2 (Forward)* | 5’-GGTGGGGTCATGTGTGTGG-3’ |
| *BCL2 (Reverse)* | 5’-CGGTTCAGGTACTCAGTCATCC-3’ |
| *FOXM1 (Forward)* | 5’-CGTCGGCCACTGATTCTCAAA-3’ |
| *FOXM1 (Reverse)* | 5’-GGCAGGGGATCTCTTAGGTTC-3’ |
| *CCND1 (Forward)* | 5’-GCTGCGAAGTGGAAACCATC-3’ |
| *CCND1 (Reverse)* | 5’-CCTCCTTCTGCACACATTTGAA-3’ |
| *c-MYC (Forward)* | 5’-TCCCTCCACTCGGAAGGAC-3’ |
| *c-MYC (Reverse)* | 5’-CTGGTGCATTTTCGGTTGTTG-3’ |
